# Feasibility and Readiness for Scaling‐Up Multiple Micronutrient Supplements in Nepal: A Qualitative Study Using the Expandnet Scaling‐Up Framework

**DOI:** 10.1111/mcn.70231

**Published:** 2026-07-17

**Authors:** Mai‐Anh Hoang, Nira Joshi, Kenda Cunningham, Usha Singh, Bibek Kumar Lal, Lila Bikram Thapa, Pooja Pandey, Rolf Klemm, Yashodhara Rana, Sudip Pokhrel, Sasmita Poudel

**Affiliations:** ^1^ Helen Keller Intl New York New York USA; ^2^ New ERA Kathmandu Bagmati Nepal; ^3^ International Food Policy Research Institute London UK; ^4^ Family Welfare Division, Ministry of Health and Food Safety Kathmandu Bagmati Nepal; ^5^ Helen Keller Intl Kathmandu Bagmati Nepal; ^6^ Johns Hopkins Bloomberg School of Public Health Baltimore Maryland USA; ^7^ Eleanor Crook Foundation Washington, D.C. USA

**Keywords:** antenatal care, ExpandNet, implementation science, maternal nutrition, multiple micronutrient supplementation, Nepal, qualitative research, scale‐up

## Abstract

Micronutrient deficiencies in pregnancy remain a critical public health issue in Nepal, where iron–folic acid (IFA) supplementation alone has not fully addressed maternal and neonatal risks. Multiple micronutrient supplements (MMS) promise broader nutritional benefits; however, real‐world guidance for their effective and equitable integration into routine maternal care is lacking. A qualitative study, guided by the ExpandNet scaling‐up framework, was conducted from February to March 2025, including 28 focus group discussions with antenatal care (ANC) providers and female community health volunteers (FCHVs) and 40 key informant interviews with policymakers at different levels of the health system. Data was analyzed thematically to identify perceived facilitators and barriers to MMS adoption, institutionalization, and scale‐up, and equitable implementation. Stakeholders viewed MMS as offering clear advantages over IFA, citing broader micronutrient coverage and fewer side effects that could improve adherence. However, they identified several system‐level requirements for successful scale‐up, including formal policy integration, sustainable financing, strengthened procurement and supply chains, and adequate storage capacity at facilities. FCHVs and ANC providers emphasized the need for clearer role definitions, improved training, and manageable counseling workloads. Persistent community‐level barriers—such as concerns about tablet size, misconceptions about fetal growth, and low trust in free medicines—were seen as risks to equitable uptake. Respondents highlighted community engagement, culturally tailored counseling, and consistent support for FCHVs and ANC providers as essential to ensuring effective and equitable MMS adoption. Stakeholders in Nepal view MMS as a promising strategy to improve maternal and newborn nutrition, but its potential depends on strong policy commitment, adequate and sustainable financing, and community‐centered implementation. Addressing product‐related concerns, strengthening counseling and trust in public services, and ensuring uninterrupted supply chains are essential to MMS scale‐up, including translating its introduction into equitable and meaningful gains in maternal and child nutrition.

## Summary

- MMS is widely preferred to IFA because it provides more nutrients and is perceived to cause fewer side effects.
- Barriers to adoption include large tablet size, persistent misconceptions and fears (e.g., excessive fetal growth), hesitancy regarding free medicines, unclear frontline roles, FCHVs perceived limited recognition of their roles and contribution within the health system, training gaps, and reliance on donor‐funded technical support.
- Successful scale‐up will require policy integration, reliable supply chains, knowledgeable and skilled providers, and community engagement.

## 1 Introduction

Micronutrient deficiencies among pregnant women remain a major global public health challenge, especially in low‐ and middle‐income countries (LMICs) like Nepal. Persistent deficits in iron, zinc, folate, and other micronutrients continue to contribute significantly to adverse maternal and newborn outcomes, including anemia, poor fetal development, preterm birth, and elevated neonatal morbidity and mortality (World Health Organization 2020). Despite decades of routine iron–folic acid (IFA) supplementation, maternal undernutrition and adverse pregnancy outcomes remain common, highlighting limitations of the current maternal nutrition strategy in Nepal (Ministry of Health and Population MoPH, New ERA, & ICF 2023).

Growing evidence now supports a transition to the UNIMMAP formulation of antenatal multiple micronutrient supplements (MMS). This formulation offers a broader nutrient profile and has demonstrated reduced rates of low birth weight, small for gestational age, and preterm birth, as well as improvements in infant growth and maternal health (Keats et al. 2019; Smith et al. 2025). Decisions on how to successfully scale‐up MMS, however, increasingly hinge not only on clinical effectiveness but also on whether health systems can deliver MMS at scale, equitably, and with high adherence. As Nepal added MMS to the National Essential Medicines List in late 2025, questions of system readiness, operational feasibility, and equitable implementation have become increasingly urgent.

From 2024 to 2025, the Government of Nepal and partners launched a series of implementation research studies to inform this transition, including the NAMASTE‐MMS cluster‐randomized trial, which evaluates maternal adherence and acceptability of MMS (in blister packs and bottles) compared to IFA in blister packs (Cunningham et al. 2025). This NAMASTE‐MMS trial, along with quantitative demonstration studies planned in other provinces of Nepal, is the only UNIMMAP‐MMS currently distributed through the public sector. Therefore, understanding stakeholder perceptions of this specific product and the routine ANC delivery model is crucial for interpreting implementation experiences and developing a national MMS scale‐up strategy.

Experience from Nepal and other LMICs shows that successful MMS adoption depends not only on clinical effectiveness but also on product acceptability, community perception, health system capacity, and the roles and motivations of frontline workers such as ANC providers and female community health volunteers (FCHVs) (Labonté et al. 2025; Sauer et al. 2025). Product attributes (e.g., tablet size, side effects, packaging), socio‐cultural beliefs, trust in public‐sector medicines, and the strength of procurement and supply chains all shape whether MMS can be delivered at scale and with high adherence.

The ExpandNet framework offers a structured lens for examining these dynamics by emphasizing how innovation attributes, user organization capacity, resource team readiness, environmental factors, and scale‐up strategies interact to influence the adoption and institutionalization of new health interventions (Simmons et al. 2009, 2010, 2011). Yet, there is limited evidence on how these factors play out within Nepal's federalized health system, where responsibilities for planning, procurement, and service delivery are distributed across federal, provincial, and local levels.

To address this gap, we applied the ExpandNet framework to explore policymakers, ANC providers, and FCHVs perspectives on the feasibility, acceptability, and system readiness for transitioning from IFA to MMS nationwide. By examining experiences across multiple tiers of the health system, this study identifies the operational requirements, perceived barriers, and enabling conditions needed to integrate MMS into routine maternal care and ensure equitable uptake at scale.

## 2 Methods

### 2.1 Study Design and Research Team

We conducted a qualitative study to explore perceived needs and anticipated challenges in transitioning from IFA to MMS in Nepal, using semi‐structured focus group discussions (FGD) and key informant interviews (KII). This study was designed to complement the NAMASTE‐MMS randomized trial, on MMS adherence and acceptability (Cunningham et al. 2025). We followed the Consolidated Criteria for Reporting Qualitative Research (CORE‐Q) and incorporated all 32 items across study design, data collection, analysis, and reporting. See Supplementary File 1: COREQ checklist.

A core team, led by NJ, supported by two supervisors with mixed‐methods expertise, oversaw all aspects of qualitative data collection and analysis. Fieldwork was conducted by six trained qualitative researchers (four women and two men) with backgrounds in public health, education, and the social sciences, including the co‐author (US).

### 2.2 Study Sites and Participant Selection

We collected data across all seven provinces of Nepal to capture perspectives from all three administrative tiers of the health system: federal, provincial, and local. Districts were purposively selected in consultation with federal and provincial authorities to capture variation in geography (Terai, hills, mountains), urban–rural mix, and implementation of ongoing MMS‐related activities. Within each selected district, municipalities and health facilities were purposively selected to reflect different facility types and to ensure inclusion of both relatively well‐resourced and more constrained settings. One district per province was purposively selected to reflect geographic and administrative diversity. Within each district, one to two municipalities and corresponding health facilities served as recruitment sites. Participants represented three stakeholder groups: policymakers, ANC providers, and FCHVs.

Purposive sampling ensured variation in roles, settings, and administrative levels. Recruitment lists were developed in collaboration with local health authorities. Eligible participants were approached in person, provided with study details, and provided with written informed consent. Although 42 KIIs were initially planned, two invitees declined due to scheduling conflicts; the others agreed to participate.

### 2.3 Data Collection Tools

The interview and FGD guides were developed to explore operational and supply‐side constraints to maternal supplementation, drawing on the WHO health system building blocks and prior evidence on antenatal iron–folic acid program bottlenecks in Nepal. Data collectors completed a 3‐day training covering study protocols, qualitative interviewing, participatory techniques (free listing, pile sorting), reflexivity, and research ethics. To ensure cultural appropriateness, we sought input from FCHVs and ANC clients during instrument development. All interview guides were piloted, translated and culturally adapted prior to data collection. See Supplementary File 2: FGD and IDI discussion guides. After data collection, the ExpandNet framework was then used primarily as an analytic and organizing lens, with emergent themes mapped to its five domains through a combined deductive–inductive approach.

**Figure 1:**
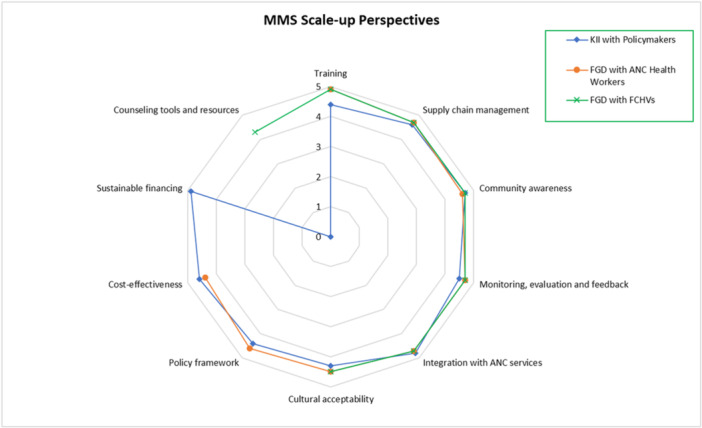
Prioritization of Key Health Systems Dimensions by Policymakers, ANC providers, and FCHVs.

In addition, during selected KIIs and FGDs, we conducted a structured prioritization exercise in which participants scored key health‐system dimensions for MMS scale‐up (e.g., training, financing, supply chain, community awareness, monitoring) on a 1–5 scale and ranked their top priorities. These scores were later synthesized in a radar chart (Figure 1) to visualize perceived readiness across the ExpandNet domains. Not all respondents scored every item, as some KIIs and FGDs completed a shortened version of the prioritization exercise due to time constraints, and in those sessions, participants were asked to focus on the domains they considered most relevant to their context.

### 2.4 Data Collection Procedures

Data collection took place between February and March 2025 and was conducted by three two‐person field teams (moderator and note‐taker). A deployment manual outlined sites, and all KIIs and FGDs were scheduled and confirmed by phone. Sessions were conducted in Nepali at health posts, municipal offices, or other private and convenient venues, with only participants and the research team present (no observers). Each KII and FGD lasted 60–90 min. All sessions were audio‐recorded on dual devices and supplemented with detailed field notes. No participants withdrew after consenting, and no repeat interviews were conducted.

Moderators used semi‐structured guides and participatory techniques‐‐included probing, free listing, and pile sorting—to elicit detailed perspectives. After obtaining written consent, participants completed a brief demographic questionnaire capturing age, gender, work experience, and organizational affiliations.

### 2.5 Ethical Considerations and Reflexivity

The study was conducted in accordance with the Declaration of Helsinki and approved by the Nepal Health Research Council (protocol #95_2025, March 29, 2024). Participation was voluntary, and written informed consent was obtained from all participants prior to data collection. Data collectors were gender‐balanced Nepali individuals with no prior relationships with participants and they were all trained in qualitative methods and research ethics.

### 2.6 Data Management and Analysis

We transcribed all KII and FGD transcripts in Nepali, translated the data into English, and checked all English transcripts against the original Nepali audio and text for accuracy. Transcripts were managed in NVivo v14. Three stakeholder‐specific codebooks were developed through a combined deductive‐inductive approach, with two independent coders applying and refining codes. Inter‐rater reliability was assessed on 10% of transcripts by MAH, NJ, and US, yielding Cohen's Kappa values of 0.61–0.80, indicating moderate to substantial agreement.

We conducted thematic analysis guided by the five domains of the ExpandNet framework, iteratively refining codes and themes until reaching thematic saturation. A hierarchical coding tree (See Supplementary File 3, Figure 1) depicts the organization of themes and subthemes. Participatory tools used during data collection—such as free listing, pile sorting, and prioritization—were incorporated into the analytic process to enrich interpretation and triangulation of findings (See Supplementary File 4 for codebooks).

Throughout data collection and analysis, the core research team held regular debriefing sessions to compare emerging insights, resolve discrepancies and ensure consistency across stakeholder groups. Direct quotations are used to illustrate key themes. Due to logistical constraints, transcripts and preliminary findings were not returned to participants for validation.

### 2.7 Ethics Statement

The following is the ethical statement included in Section 2.4 of the Main Text of the manuscript: “The study was conducted in accordance with the Declaration of Helsinki and approved by the Nepal Health Research Council (protocol #95_2025, 29 March 2024). Participation was voluntary, and written informed consent was obtained from all participants.”

## 3 Results

In this section, we synthesize stakeholder perspectives on MMS, with particular attention to how the transition from IFA to MMS interacts with Nepal's health systems' strengths and gaps. Findings are drawn from the following participants:

- Policymakers: 40 in‐depth KIIs across federal, provincial and municipal tiers;
- ANC providers: 14 FGDs with 100 ANC providers from sampled facilities; and
- FCHVs: 14 FGDs with 103 FCHVs who report up to the sampled facilities.

Findings are organized using the five domains of the ExpandNet framework—innovation attributes, user organization attributes, resource team attributes, environmental attributes, and scale‐up strategy—to capture the multi‐level factors shaping MMS adoption and institutionalization. Our findings draw insights from KIIs and FGDs, with quotations illustrating convergent and divergent perspectives across stakeholder groups.

### 3.1 Innovation Attributes

According to the ExpandNet framework, certain features of a new intervention—such as whether it seems better than what came before, whether people trust it, and whether they find it acceptable—shape how widely it is used and scaled up. In Nepal, these features influenced how providers, policymakers, and communities viewed MMS relative to IFA.

#### 3.1.1 Relative Advantage and Perceived Effectiveness

Across stakeholder groups, MMS was widely viewed as a clear improvement over IFA. Participants emphasized its broader nutrient profile, potential to address multiple deficiencies, and generally better tolerability, including fewer reports of gastrointestinal discomfort, nausea, and vomiting among women currently receiving MMS through these implementation research activities (NAMASTE‐MMS and provincial demonstration studies). MMS was seen as serving the same core purpose as IFA—preventing anemia and supporting maternal nutrition—but with added value for fetal growth, and long‐term child health when women adhered consistently. Although a few providers noted that women with anemia might still need additional iron or tailored management, these concerns did not overshadow the overall perception of MMS as a better option.

Local‐level providers, such as auxiliary nurse midwives (ANM) and FCHVs, tended to focus on visible benefits and women's day‐to‐day experiences with tablets. “Previously, only iron and folic acid tablets were provided… Now MMS not only gives immediate benefits, but we see it having long‐term positive effects for mother and baby” (FGD, health worker). Higher‐level officials tended to focus on the product composition and programmatic implications, while also raising questions about iron adequacy in settings where anemia is a severe public health problem. As one provincial health official explained, “IFA only includes iron and folic acid… MMS contains 15 types of vitamins, so it can address multiple deficiencies, not just anemia” (KII, provincial health official). Another respondent noted, “Compared to iron and folic acid, MMS has fewer side effects… women are more likely to accept it and continue taking it” (KII, Family Welfare Division [FWD] official), while others cautioned that “it might be a bit difficult to convince some people at first, because the iron content in MMS is lower and in our community many mothers are anemic; some may still need extra iron” (KII, FWD official). Similarly, an ANM noted that, compared to iron alone, “the growth and development of the child is also better… It is more effective than iron” (FGD, ANM).

#### 3.1.2 Acceptability and Product Characteristics

Acceptability was shaped by both product characteristics and prevailing beliefs about medicines given in pregnancy. Tablet size—especially for blister‐packed MMS—was a common concern among providers and pregnant women, with some ANM explaining that “the tablet is large and difficult to swallow… we advised them to break it” (FGD, ANM). At the same time, stakeholders described persistent fears familiar from IFA, such as worries that tablets might cause “larger babies,” and noted that a broader lack of confidence in free medicines from public health facilities could discourage uptake of both IFA and MMS. As one community volunteer observed, “There is a lack of trust when something is free… they doubt whether the medicine will work” (FGD, FCHV). Despite these concerns, positive personal experiences with MMS helped some women overcome initial hesitations about tablet size and free medicines. One FCHV recounted that a woman who had previously felt nauseous when taking iron tablets “hasn't had any issues” since taking MMS during her current pregnancy, which supported her continued use over time (FGD, FCHV).

### 3.2 User Organization Attributes

In the ExpandNet framework, the *user organization* means the parts of the health system and the people responsible for taking up and maintaining new practices. In Nepal's federalized system, this includes coordination among federal, provincial, and local governments as well as health facilities and frontline workers. The findings emerged in two interlinked dimensions: implementation capacity and leadership commitment.

#### 3.2.1 Implementation Capacity, Logistics, and Workload

Decentralized procurement and planning introduced both flexibility and fragmentation. Provincial and local respondents described ongoing challenges with forecasting, managing stock, and handling logistics which affected antenatal supplements. Municipal officials and facility staff noted that limited human resources and weak stock monitoring contributed to both stock‐outs and over‐procurement. As one provincial logistics representative explained, “our strong recommendation is to improve forecasting… we over‐purchase and supplements expire” (KII, provincial logistics representative).

These system‐level challenges were particularly evident after procurement responsibilities shifted from district to municipal authorities. A facility manager described how “the supply problem is primarily due to an issue at the central level… we need the supplies to come from the center, but they are coming from the municipality… when we go, either the items are not available, or they haven't been delivered yet, and we end up in a difficult situation” (FGD, public health officer in‐charge).

Facilities also reported constraints in storage capacity for antenatal supplements, particularly for bulkier MMS blister packs compared with IFA. ANM described “a storage issue,” explaining that “MMS requires more space because each bottle needs its own place, and there's not enough space in the drawers… the current storage setup isn't enough” (FGD, ANM). Some respondents suggested that alternative, more compact packaging could be more space‐efficient and easier to organize for routine MMS distribution.

Providers noted that counseling, screening, and documentation for antenatal supplements were already time‐consuming under IFA, and that introducing MMS initially added to counseling demands because it was a new product and women had questions about how it differed from IFA. One midwife observed that “of course, the workload increases… counseling each one takes about half an hour… when working alone, it's sometimes impossible to manage everything” (FGD, ANM). Similarly, a facility manager anticipated that “healthcare workers will definitely face an increased workload because, although we were *supposed* to explain the benefits and risks of IFA, in practice this was often rushed or skipped; with MMS, we now need to explain much more—its benefits, risks, and provide detailed information to each person, which consumes a lot of time” (KII, public health officer in‐charge). Together, these perspectives show that while MMS is being layered onto existing antenatal systems, gaps in local procurement capacity, storage, and workload management must be addressed to support effective and sustainable scale‐up.

#### 3.2.2 Leadership, Accountability, and Resource Allocation

Respondents at all levels stressed that clear leadership and accountability were crucial for sustaining antenatal supplementation and for managing a transition to MMS. While many noted active engagements from municipalities and provinces, they emphasized that “the leadership at the local level plays a significant role… they can allocate resources from the municipal budget” (KII, federal logistics official). At the same time, participants called for stronger federal stewardship — particularly for centralized procurement, clear guidelines, and dedicated budget lines for MMS, as currently exists for IFA. As one municipal respondent suggested, “for a smooth transition, we recommend the federal government handle procurement centrally, just like IFA” (KII, municipal health official). Participants emphasized that clearly defined roles across federal, provincial, and local levels are essential to prevent implementation gaps, procurement delays, and financing uncertainties, and to ensure long‐term sustainability, quality assurance, and equitable supply across provinces.

Resource constraints—financial, infrastructure, and workforce—were consistently cited as bottlenecks to scaling up MMS beyond pilot sites. A senior official noted that “our current budget constraints prevent us from expanding distribution at this time” (KII, senior official, FWD). Overall, Nepal's decentralized structure offers opportunities for local adaptation but also exposes disparities in institutional capacity; respondents highlighted that fragmented supply chains, storage and staffing constraints, and evolving FCHV roles could hamper MMS implementation unless accompanied by coordinated leadership, clear role definitions, and predictable financing for antenatal supplementation.

### 3.3 Resource Team Attributes

Under the ExpandNet framework, the *resource team* is the group of people and organizations responsible for taking a new intervention to scale. In Nepal, this includes policymakers, ANC providers, and FCHVs, whose competence, motivation, and coordination determine the trajectory of MMS scale‐up. In our analysis, four themes emerged: how well different levels of government coordinate, the quality and reach of training, the strength of counseling and communication, and how clearly FCHV roles are defined and supported.

#### 3.3.1 Coordination Roles and Strategic Leadership

Policymakers emphasized the need for stronger coordination across federal, provincial, and local levels for antenatal supplementation, including MMS. They said that, although people know their roles, there are still communication gaps, uneven supervision, and limited joint planning, noting that “there needs to be a more effective coordination mechanism among the three levels of government… it's a matter of everyone's right to access services” (KII, provincial policymaker). These gaps suggest that, even though the main structures and actors for MMS scale‐up already exist, a shared strategic vision and routine mechanisms for collaboration are still needed, particularly for integrating MMS into existing IFA platforms.

#### 3.3.2 Technical Expertise and Training Needs

Respondents agreed that training for health workers is crucial for the successful scale‐up of antenatal supplements, but is often under‐resourced and insufficient. They described training as short, cascade‐style training, and not always reaching all staff or giving enough time to practice or ask questions. Participants stressed that, as MMS is rolled out nationally, training should not focus only on ANM but also include other frontline cadres who provide antenatal care and supplementation follow‐up. They felt that other frontline health workers and health facility staff also need training to assist with ANC follow‐up visits and that “training FCHVs for outreach will be highly effective” (FGD, health worker; FGD, ANM). They also suggested involving facility management committees, private providers, and traditional healers so that messages and referrals are more consistent. For example, one provider recommended that “private hospital staff should also be trained to understand MMS replaces iron supplements,” while another emphasized that “orientation for shamans (traditional healers) is important to align traditional and medical advice” (FGD, ANM; FGD, health worker).

Respondents also stressed that the MMS scale‐up will require more than just one‐off training. Participants emphasized that training should cover both technical content (e.g., MMS composition, indications, and side‐effect management) and practical counseling skills (e.g., addressing myths about fetal growth, explaining differences from IFA, and tailoring messages to low‐literacy audiences). They called for ongoing supportive supervision and regular refresher sessions to maintain high quality, with some suggesting “refresher training… every 90 days, involving both service providers and beneficiaries” (FGD, health worker). In ExpandNet terms, the strength and reach of training and supervision are key to resource‐team readiness, and relying only on short, cascade‐style training without follow‐up could limit the system's ability to sustain MMS at scale.

#### 3.3.3 Visit Time and Workload Constraints

Adherence to MMS depended not only on supply but also on ANC providers' ability to spend enough time with each client, which was often squeezed by limited time and heavy workloads. Because MMS was a new product, providers reported that it required “a lot more” explanation than IFA and that “MMS requires a lot of time… managing other cases is hard” (FGD, ANM; KII, provincial health worker). Busy clinics and staff shortages meant that even motivated providers could not always give each woman clear, tailored information on why MMS was needed, how long to take it, and what to expect. As one midwife reflected, “if we had fewer patients, we could take the time to explain.” These accounts show that counseling was constrained primarily by short consultation times and competing demands during ANC visits, limiting opportunities to reinforce MMS messages and troubleshoot side‐effects. Programmatically, this points to the need to reduce provider workload where possible and to integrate simple MMS prompts into routine ANC workflows so that key messages can be delivered efficiently within existing visit lengths.

#### 3.3.4 Role Clarity, Support, and Recognition

FCHVs described being unsure about their role in antenatal supplementation. They knew that government rules now place most tablet distribution in facilities, but they were not clear what this meant for their own tasks—such as home follow‐up, tracing women who had stopped, and doing community awareness—which left some feeling less confident and less visible in the system. As one FCHV said, “we are still confused about whether we are supposed to distribute this pill or not” (FGD, FCHV).

As unpaid volunteers, many also spoke about having little financial or other recognition. Several noted that “we don't receive any financial support… we can't work on an empty stomach” (FGD, FCHV), showing how economic pressures affect their ability to take on extra work like MMS. These views suggest that scaling up MMS effectively will require clearly defining and communicating FCHVs' roles in community awareness, early pregnancy identification, and adherence follow‐up, as well as providing appropriate recognition and incentives to keep them engaged.

### 3.4 Environmental Attributes

In the ExpandNet framework, the *environment* covers outside factors—such as policies, politics, social norms, and the broader health system—that can help or hinder scale‐up. In Nepal, two parts of this environment stood out for MMS: the policy setting and political priorities.

#### 3.4.1 Policy Environment

Recent updates to the National Medical Standard for Maternal and Newborn Care (Volume III) and the inclusion of MMS on the National Essential Medicines List have created an initial policy foothold for MMS. Respondents noted that these national signals, however, were not yet matched by clear operational guidance, dedicated budget lines, or harmonized implementation plans at provincial and local levels. Local governments had some discretion to prioritize maternal nutrition within their broader health plans, but engagement and resource alignment for MMS varied widely, with one district respondent noting that “local government involvement is indispensable for effective implementation at the grassroots level” (FGD, public health official).

Some providers interpreted the placement of MMS within existing maternal health programs as an opportunity to secure resources for future rollout. As one ANM suggested, MMS “falls under the Safe Motherhood program, so they should allocate budgets accordingly” (FGD, ANM), highlighting how frontline staff look to program labels and budget headings as cues for prioritization. From an ExpandNet perspective, these mixed signals indicate partial policy coherence: the foundational documents for MMS scale‐up are in place, but sustained budget allocations, detailed operational guidelines, and alignment between federal, provincial, and local plans are needed to institutionalize MMS within routine antenatal care and avoid fragmented implementation.

#### 3.4.2 Political Environment

Political will was described as highly reactive, often triggered by international endorsements rather than local leadership. Several respondents perceived that once international bodies formally endorsed MMS, it would be reflected in national standards such as the Essential Medicines List and political resistance would be limited; active prioritization of MMS in annual plans and budgets, however, would still be required at each level of government. As one provincial official observed, “once the UN recommends it (MMS), the government will implement it without significant issues” (KII, provincial official).

Overall, the political environment was seen as cautiously supportive but not yet fully mobilized: international and national endorsements have opened a window for MMS, but translation into concrete commitments—such as earmarked financing, inclusion in local annual work plans, and sustained attention in health sector reviews—will determine whether this opportunity leads to durable scale‐up. Local‐level respondents emphasized that decentralized authority and budgets could either accelerate or stall implementation, depending on how aware and engaged municipal leaders are. One ANM noted that “since local governments have taken responsibility, there is more focus on proper distribution… local governments have their own budgets, so they can purchase and distribute it if needed,” while a facility manager stressed that “if the local government is aware of this, the acceptance rate will increase significantly… if this comes from the local government, it will be more widely accepted” (FGD, ANM; FGD, public health officer in‐charge).

#### 3.4.3 Socio‐Economic and Cultural Context

Cultural norms, gender dynamics, and health literacy strongly influence uptake. Traditionally, mothers‐in‐law and husbands held decision‐making authority; there is some concern that their fears and misconceptions (e.g., MMS leads to “larger babies”) may cause them to discourage pregnant women from taking MMS. The need for locally tailored counseling that involves key family decision‐makers was underscored. As one community volunteer explained, “whatever their mother‐in‐law decides is usually what they follow,” while a provincial coordinator noted that “there is a notion… that vitamins during pregnancy lead to larger babies, making childbirth more difficult” (FGD, FCHV; FGD, provincial health coordinator). These findings suggest that acceptance is shaped not only by women's education, socio‑economic status, religion, and the perceived “status” of free supplements but also by how MMS is presented and packaged within existing family power dynamics and beliefs.

#### 3.4.4 Health Sector Context

Women from remote mountainous areas, poorer households, and marginalized groups were viewed as less likely to complete the recommended number of ANC visits and may miss opportunities to receive IFA or MMS through routine contacts. Participants highlighted that difficult terrain, seasonal road access, and limited facility density continue to make it challenging to reach some populations, despite national gains in coverage. ANC providers emphasized that “due to Nepal's geographical landscape, it might be difficult to reach remote areas,” and that in many under‐served communities “health‐related services haven't reached them directly” (FGD, ANM; FGD, provincial health worker). These suggest that frontline providers see persistent geographic and socio‐economic inequities that constrain who can actually benefit from MMS introduced through routine ANC, even where national coverage indicators appear high.

### 3.5 Scale‐Up Strategy

In the ExpandNet framework, the *scale‐up strategy* is the plan for how a new intervention spreads to more places (horizontal scale‐up) and becomes part of routine national systems (vertical scale‐up). For MMS in Nepal, both are important: coverage needs to expand across provinces, while policies, budgets, and responsibilities are built into national and local systems. Stakeholders identified four factors that will be especially important for success: clear planning and coordination, strong communication and advocacy, secure and sufficient funding, and regular monitoring and evaluation.

#### 3.5.1 Strategic Planning and Coordination

Stakeholders emphasized that effective MMS scale‐up will require phased implementation, clearly delineated roles for all actors involved in procurement, supply, and service delivery. As one health coordinator noted, “responsibility must be divided clearly… who is in charge of procurement, supply, and implementation” (FGD, health coordinator), underscoring the need for explicit role definition to avoid gaps or duplication. Centralized federal guidance, combined with strong multisectoral and local coordination, was viewed as crucial for achieving equity and consistency across provinces.

In addition, stakeholders underscored the need for sustained capacity building at the local and provincial levels to ensure a resilient and adaptive health system as MMS efforts expand. A district respondent emphasized that “when planning this program before its implementation, the local level and the province should be empowered to build their capacity” (FGD, public health official), highlighting that planning for MMS must include investments in subnational systems, not only in products and protocols.

#### 3.5.2 Dissemination and Advocacy

Community‐level communication, led by trusted people such as FCHVs and mothers' groups, was seen as essential for generating demand for MMS and overcoming skepticism. Provincial stakeholders emphasized that “FCHVs… should be made the agents of awareness. Since they are deeply integrated into the community… women trust them” (KII, provincial policy official), highlighting their unique position to translate technical guidance into locally resonant messages. Monthly mothers' group meetings were described as a particularly important platform for repeated contact and discussion. As one coordinator noted, “the strongest and most consistent channel is the mothers' group meeting… every month” (FGD, health coordinator). In addition to these face‐to‐face channels, participants pointed to local radio, FM stations, and messages in local languages as effective ways to reach different groups and repeat key points about MMS, especially where literacy is low or access to facilities is limited.

#### 3.5.3 Cost and Resource Mobilization

Concerns about MMS's higher per‐unit cost frequently came up but were often tempered by recognition of its added value compared with IFA. Provincial officials noted “a concern that MMS might be more expensive… because it contains a broader range of nutrients” (KII, provincial official), yet many stakeholders emphasized that predictable budget allocation and a continuous supply would be more decisive than unit price alone for sustainability. As one coordinator explained, “to expand MMS to all health facilities, we need to allocate some budget… if financial support is strong, it will be easier” (FGD, health coordinator), underscoring the central role of domestic financing and resource mobilization, including for FCHV incentives.

#### 3.5.4 Monitoring and Evaluation

Integrated, digital monitoring systems—using platforms such as DHIS2, HMIS, or LMIS—were widely cited as essential for transparency, real‐time troubleshooting, and continuous learning. A provincial respondent stressed that “once MMS is implemented, the reporting system in DHIS2 must be integrated from the start” (KII, provincial health official), highlighting the importance of designing indicators and data flows before scale‐up. Others emphasized that “periodic reviews are essential… to discuss any gaps and challenges” (FGD, health officer), pointing to regular review meetings across levels as a key mechanism for using data to improve implementation.

Overall, Nepal's MMS scale‐up strategy shows a promising outlook for horizontal expansion but remains in the early stages of vertical institutionalization. Formal policy inclusion, stable financing, and robust digital monitoring platforms will be essential to embed MMS within national systems; achieving both breadth and depth of coverage will require continued federal leadership, strong intergovernmental coordination, and sustained community engagement to transform a promising pilot into a durable public health program.

### 3.6 Prioritization Exercise and Radar Chart Findings

The structured prioritization exercise (Figure 1) provided a participatory snapshot of the system's readiness to scale MMS across the ExpandNet domains. Stakeholders (policymakers, ANC providers, and FCHVs) together identified the operational and system elements essential to transitioning MMS from pilot to national‐scale‐up. All three groups assigned the highest importance to training, sustainable financing, supply chain management, integration with ANC services, community awareness, and robust monitoring and evaluation systems, with mean scores approaching 5 on a scale of 1–5.

FCHVs placed particular emphasis on community awareness and engagement, reflecting their frontline view of how local trust and social norms affect uptake. Sustainable financing, sufficient time and tools for counseling, and capacity‐building (training and supportive supervision) also consistently emerged as top priorities, suggesting broad agreement that system‐level investment and ongoing support for frontline workers are central to effective and fair implementation. Cost‐effectiveness and wider policy frameworks were rated somewhat lower but still important, indicating that stakeholders were primarily focused on practical readiness and direct support for service delivery and adherence.

### 3.7 Synthesis of Findings

Table 1 brings together the main implementation challenges and opportunities for MMS scale‐up as reported by each stakeholder group, organized using the ExpandNet domains. It is based directly on KIIs and FGDs and gives a brief, practical overview of ongoing barriers (such as complex supply chains, heavy staff workloads, and community fears and misconceptions) and key enablers (including committed local leadership, policy integration, and active use of mothers' groups and digital tools). By showing issues at different levels of the system and how they interact, the table can help guide national planning and program design.

**Table 1:** Barriers and enablers for MMS scale‐up in Nepal, by ExpandNet Domain and stakeholder group.

ExpandNet domain	Policymakers	ANC providers	FCHVs
Innovation attributes	Barriers: Skepticism about lower iron content in MMS in high‐anemia settings. Enablers: Shared perception that MMS is “more complete” and superior to IFA (broader nutrient profile, fewer side effects, perceived benefits for maternal strength and fetal growth).	Barriers: Large tablet size and swallowing difficulty, occasional nausea or dizziness. Enablers: Improved tolerance, fewer side effects compared to IFA.	Barriers: Large tablet size and swallowing difficulty, occasional nausea or dizziness. Enablers: Women's positive experiences once they switch from IFA to MMS, and generally positive views of MMS packaging.
User Organization (Health System)	Barriers: Fragmented, decentralized procurement and logistics; lack of federal guidance; partial policy coherence. Enablers: Existing policy platforms for integrating MMS.	Barriers: Heavy workload, inadequate staff and storage, and documentation burden. Enablers: Strong intrinsic motivation to support women if provided with clear protocols, practical training, and job aids.	Barriers: Ambiguity regarding FCHV responsibilities and diminished sense of purpose or community recognition, stemming from being excluded from MMS distribution activities Enablers: High trust as community mobilizers and educators.
Resource team	Barriers: Weak inter‐government coordination, reliance on project‐based training. Enablers: Growing policy interest at national and subnational levels; technical partnerships.	Barriers: Rushed, cascade‐style training with limited time for practice; lack of refreshers and materials undermining counseling confidence. Enablers: Strong demand for hands‐on, interactive training, refresher sessions, and supportive supervision.	Barriers: Lack of clear role definition, compensation, and formal recognition. Enablers: Motivation rooted in community respect, service ethos, and desire to contribute to MMS rollout.
Environment	Barriers: No dedicated budget line for MMS, inconsistent prioritization across municipalities, and donor dependence. Enablers: Recent inclusion of MMS in national standards and lists, and scope for local adaptation under decentralization.	Barriers: Stock‐outs of supplements, difficult terrain, seasonal road access, and cross‐border movement limiting consistent ANC contact. Enablers: Facility‐level champions and managers actively problem‐solving to maintain services.	Barriers: Low literacy, elder‐ and husband‐dominated decision‐making, and persistent misconceptions about supplements. Enablers: Access to mothers' groups, FCHV networks, and local/religious influencers who can shape norms.
Scale‐up Strategy	Barriers: Weak resource mobilization and fragmented monitoring systems; political will is often reactive to external endorsements. Enablers: National pilots, phased planning discussions, and emerging use of digital platforms (DHIS2, HMIS, LMIS) for MMS.	Barriers: Time burden for individualized counseling; lack of standard MMS counseling materials and clear guidance on differences from IFA. Enablers: Strong desire for supervision, job aids, and regular review meetings to use data for course correction.	Barriers: FCHVs noted unclear expectations around MMS‐related tasks and the absence of incentives. Enablers: Mothers' group meetings, local radio/FM, and FCHV‐led outreach seen as trusted channels for demand generation.

## 4 Discussion

This study, which covered all seven provinces and multiple levels of Nepal's health system, is the first national assessment of the country's readiness to transition from IFA to MMS in routine antenatal care. We use the ExpandNet framework to qualitatively examine how policies, health system structures, and community factors interact in a federal setting. We found that stakeholders widely see MMS as better than IFA and potentially more beneficial for mothers and newborns, but that these benefits depend on solving practical problems in financing, procurement, supply chains, and frontline counseling.

Our findings show how implementation is shaped by a mix of product attributes, system constraints, and beliefs in families and communities. Large tablets, concerns about lower iron content, and doubts about free medicines combined with fragmented supply chains, uneven local capacity, and family decision‐making to influence acceptability and adherence. By mapping these issues to the ExpandNet domains, we highlight that even a clinically superior product like MMS will have a limited impact if it is introduced into weak delivery systems.

In Nepal's federal context, we add new evidence on how decentralization affects service delivery. Local governments can tailor MMS delivery, but differences in leadership, budgeting, and logistics mean that counseling quality, supply continuity, and follow‐up would vary across areas. This aligns with work on Nepal's Multi‐sectoral Nutrition Plan, which found that federalization has improved horizontal coordination and local action but disrupted vertical coordination between federal, provincial, and local levels (Eissler et al. 2026). Our results suggest that MMS scale‐up must address procurement, supply chain, and counseling gaps while also investing in clearer inter‐governmental roles and accountability, stronger multi‐level coordination platforms, and better feedback loops between central, provincial, and local levels.

Evidence from Ethiopia and other African countries has underscored that the success of MMS and other maternal health and nutrition interventions depends on reliable procurement, continuous supply, and strong supervision and training of frontline workers, alongside context‐specific community engagement to address fears and misconceptions (Berhanu et al. 2025; Nishimwe et al. 2021). These practical insights emphasize that achieving meaningful MMS coverage depends not only on acceptability and superior product attributes but also on systematic investment in supply, coordination, and workforce capacity. For example, a pilot implementation of MMS in Rwanda reported high ANC attendance and MMS adherence but identified limited patient‐centered counseling, financial barriers, access constraints, and product stock‐outs as key challenges to address in moving from pilot to national scale‐up (Pastori et al. 2026). Similar implementation themes have been reported in Asian settings, where sustainable financing, stronger integration of community health worker programs into formal health systems, and clear role differentiation and support for community health workers are highlighted as prerequisites for effective scale‐up of primary healthcare interventions (Mark Mwenda 2024).

In Cambodia and Bangladesh, prior studies have shown that adherence to prenatal supplements is heavily influenced by the quality of counseling, women's trust in public services, family decision‐making, and socio‐cultural beliefs (Labonté et al. 2025; Nguyen et al. 2017, 2018; Sanghvi et al. 2023). Our study is consistent with these findings and adds detail from Nepal on how similar behavioral and social factors intersect with decentralized procurement and service delivery. These patterns mirror broader evidence from Nepal that harmful gender norms, restricted mobility, and limited awareness can constrain women's ability to act on ANC advice, even when services and tablets are available (Joshi et al. 2024; Morrison et al. 2021; Paudyal et al. 2022). This literature and our findings underscore the need for locally tailored counseling that engages mothers‐in‐law and husbands, addresses fears about “larger babies,” and presents MMS in ways that are trusted and dignifying, including attention to packaging and the perceived “status” of free supplements.

Finally, equity in reach remains a concern. Nepal has achieved high overall ANC coverage and IFA distribution, with recent DHS data indicating that most women receive ANC from a skilled provider and many complete at least four visits, yet important inequities persist (Ministry of Health and Population MoPH, New ERA, & ICF 2023) Our participants perceived that women from remote mountainous areas, poorer households, and marginalized groups are less likely to complete the recommended number of ANC visits and thus may miss opportunities to receive MMS through routine contacts, due to difficult terrain, seasonal road access, and limited facility density. This suggests that frontline providers see persistent geographic and socio‐economic inequities that constrain who can actually benefit from MMS introduced through routine ANC, even where national coverage indicators appear high.

MMS scale‐up in Nepal appears both timely and feasible, provided program readiness is matched by system readiness. Our findings point to four linked requirements for institutionalizing MMS—sustainable financing, integration into ANC platforms, better supported frontline workers (including FCHVs), and meaningful community engagement. In ExpandNet terms, Nepal seems well placed for horizontal expansion, but full integration into the health system will depend on sustained domestic commitment, reliable budget lines, clear roles across levels of government, and concrete steps to address cultural concerns, strengthen providers' counseling, and improve routine data and feedback systems.

### 4.1 Strengths and Limitations

This study has several strengths. We drew insights from all seven provinces by collecting data in each province from a wide range of stakeholders, from national policymakers to frontline workers, and used participatory and prioritization tools to deepen and triangulate our findings. At the same time, there are limitations. We focused on policymakers and frontline providers and did not include pregnant women or their families in this study. Although complementary research linked to the NAMASTE‐MMS trial and other implementation work in Nepal has examined end users' experiences and the acceptability of MMS, this design limits our ability to capture lived experiences, intra‐household decision‐making, and equity concerns from their perspective.

Moreover, given participants' professional roles and awareness of national policy momentum around MMS, our findings may also be affected by social desirability bias, with some respondents potentially overstating support for MMS or under‐reporting implementation challenges. Power asymmetries between national‐level decision‐makers and frontline workers, and between supervisors and staff within facilities, may likewise have shaped which gaps in leadership, financing, and supervision were raised and how they were framed. Some remote or marginalized groups may be under‐represented because of logistical and resource constraints during recruitment, and transcripts were not returned to participants for validation, which may limit the completeness of our interpretations.

Finally, although we analyzed the transcripts with an equity lens, the study was designed to fully assess how MMS scale‐up could differentially affect regions or socio‐economic groups; there is a risk that introducing MMS primarily through facility‐based ANC could inadvertently widen gaps for women who already face barriers to ANC access. Despite these limitations, the study offers contextualized, practical insights for Nepal and other countries with decentralized health systems that are considering a transition to MMS.

## 5 Conclusion

In summary, Nepal's transition from IFA to MMS in routine ANC is timely and broadly supported, but its success will depend on closing critical gaps in financing, supply, and frontline counseling. Our findings point to four cross‐cutting priorities for institutionalizing MMS at scale: securing sustainable funding, integrating MMS within existing ANC platforms, strengthening and recognizing frontline workers, and engaging communities in ways that respond to local beliefs and constraints. Addressing these priorities in a coordinated way can help ensure that MMS reaches pregnant women consistently and equitably across Nepal's decentralized health system.

## Author Contributions

M.A.H., K.C., and Sasmita Poudel (S.P.) conceived and designed the study and oversaw project administration. N.J., U.S., and S.P. conducted the training of data collectors, coordinated data collection and managed the study database. M.A.H., N.J., U.S., and S.P. conducted the statistical analyses and interpreted the data. M.A.H., N.J., and S.P. drafted the initial version of the manuscript. N.J., U.S., S.P., Y.R., P.P., R.K., B.L., L.B.T., and Sudip Pokhrel (S.Po) coordinated study activities and contributed important insights during study execution and manuscript preparation. All authors revised the manuscript critically for important intellectual content, approved the final version for submission, and agreed to be accountable for all aspects of the work.

## Conflicts of Interest

YR and SPo, who are employed by Eleanor Crook Foundation, provided technical guidance on study design and data collection. NJ, US, and KC report a relationship with Helen Keller Intl that includes consulting support and travel reimbursement. All other authors report no conflicts of interest.

## Supporting information


Supporting File 1

Supporting File 2

Supporting File 3

Supporting File 4

## Data Availability

The data that support the findings of this study are available on request from the corresponding author. The data are not publicly available due to privacy or ethical restrictions.
