# Supplementary material for: Feasibility and Readiness for Scaling‐Up Multiple Micronutrient Supplements in Nepal: A Qualitative Study Using the Expandnet Scaling‐Up Framework
Source: Matern Child Nutr. 2026 Jul 17;22(3):e70231. doi: 10.1111/mcn.70231 (PMC13377524; doi:10.1111/mcn.70231)
Supplement: Supplementary file 2 — Supporting File 2 [file MCN-22-e70231-s004.docx]

# **Supplementary File 2: Key Informant Interview and Focus Group Discussion Tools**

## 2a. Key Informant Interview Guide for Policy Makers

### Goal:

To gather feedback and recommendations by actively engaging with provincial and municipal policymakers to understand their perspectives, gain valuable insights, and develop effective strategies to ensure a smooth and successful transition from Iron Folic Acid (IFA) to Multiple Micronutrient Supplements (MMS) in the provinces of Nepal.

### Research Questions:

1. What sensitization and awareness-raising strategies could support the widespread adoption of MMS among key provincial stakeholders?
2. What strategies can ensure a smooth transition at the municipal level and at the provincial level?

### Respondents:

- Policy makers from the federal, level in each of the seven provincial demonstration projects
- Provincial level policymakers in each of the seven demonstration projects
- Local-level policymakers in each of the seven demonstration projects

#### Duration: Approx. 60-90 minutes

#### Introduction (5 minutes):

- Welcome and thank participants for their time
- Explain the objective: To understand their approach and recommendations regarding MMS adoption awareness and ensuring an effective transition at the regional level
- Assure confidentiality and anonymity
- Obtain written informed consent

### Part 1: Raising Sensitivity and Awareness (25 minutes)

#### Understanding Stakeholder Perceptions:

"Let's start by discussing your thoughts on MMS and its implementation in your community."

1. Do you know about MMS being launched? Or any activities of MMS?

1. What do you think are the top 3 reasons we should transition from IFA to MMS?

3. In your experience, what are the most common questions or concerns you encounter in your federal/province/municipality regarding MMS during pregnancy?

4. Can you identify any potential cultural, social, or economic factors that might influence the implementation of MMS in your area?

5. Are there any misconceptions about MMS within your federal/province/municipality?

- Probe: If so, how can we effectively address each one?

1. Are there any concerns about MMS within your federal/province/municipality?
2. Consider the different socioeconomic realities in your community, how might economic factors influence the success of the MMS transition?

- Probe: what about access to information—how will it influence success of the MMS transition?

#### Effective Awareness Strategies

"Now, let's explore some strategies for raising awareness about MMS."

8. What are some practical ways to raise awareness of MMS and its benefits among key stakeholders such as government officials, community leaders, and health professionals?

9. In your opinion, what are the advantages and disadvantages of different communication channels (e.g., traditional media, social media, training programs, workshops) for reaching relevant audiences?)

Probe: How should strategies differ by social economic classes? Different cultures and Tribes?

10. What roles can key community stakeholders can/should play to help support MMS scale-up?

Probe: Mayor? Ward chair? Religious leader?

11. Where do you think resources could be most effectively allocated for awareness-raising campaigns in your community?

Probe: Community festivals? Celebrations; Health Campaigns?

#### Stakeholder Engagement and Support:

"Let's discuss the importance of stakeholder engagement in this transition process."

12. Who are some of the key provincial/municipal stakeholders whose support is critical for a successful transition to MMS?

- Probe: Mayor? Local leaders (ward chairperson)? Teachers? Religious leaders?, FCHVs, Health officials? Local health workers

13. What strategies can be used to actively engage these key stakeholders in the MMS transition process?

14. How can we effectively leverage their influence and resources to promote and support MMS within the province?

15. Can you suggest any names of NGOs, private organizations, businesses, or associations that can help scale MMS?

- Probe: In what capacity can they help scale?

### Part 2: Ensuring Smooth Transitions (25 minutes)

#### Logistical Considerations:

"Let's now focus on the practical aspects of implementing this transition."

16. What potential challenges do you foresee at the provincial/municipal/district level regarding the supply chain, storage, and distribution of MMS?

17. Are there any specific challenges that might be unique to rural or hard to reach areas in your province?

18. Can you suggest some possible solutions or best practices to ensure efficient and sustainable access to MMS across the province?

19. How can we optimize coordination between national, provincial, and municipal authorities to address potential barriers?

#### Training and Capacity Building:

"Now, let's discuss the training and resources needed for this transition."

20. What specific training and resources do you think healthcare providers and other relevant health staff need to effectively counsel and advocate for the use of MMS?

21. Are there any additional training or resources that government workers (outside of health workers) might need to support this transition?

22. Can you suggest some strategies at the provincial and municipal levels to ensure a well-trained workforce ready for this transformation?

23. How can we ensure effective communication and collaboration between different levels of the healthcare system (federal, provincial, municipal, district) during this transition process?

#### Monitoring and Evaluation:

"Finally, let's consider how we can track and improve the transition process."

24. What mechanisms would you recommend for monitoring and evaluating the MMS transition at the provincial/municipal/district level?

25. How can we identify challenges and adapt strategies based on real-time data and feedback?

26. What platform would you recommend for regular stakeholder collaboration?

- Probe: What platform would you recommend for regular feedback from the federal/provincial/district level?

### Part 3: Prioritization

Remember to consider the cultural sensitivities and political dynamics of the province/municipality, encourage open dialogue, and adapt the discussion flow based on the participants' responses and interests. You may also want to provide visual aids or resources to facilitate understanding and encourage more engagement.

Please rate your priority on a scale of 1-5, where 1 is "Not at all important," and 5 is "Extremely important."

| Key health system dimensions | 1 | 2 | 3 | 4 | 5 |
| --- | --- | --- | --- | --- | --- |
| Health worker training: Training adequacy and capacity building for healthcare providers. | ⚪ | ⚪ | ⚪ | ⚪ | ⚪ |
| Supply chain management: Addressing stockouts, storage, and distribution challenges | ⚪ | ⚪ | ⚪ | ⚪ | ⚪ |
| Community awareness: Raising public knowledge about MMS benefits. | ⚪ | ⚪ | ⚪ | ⚪ | ⚪ |
| Policy framework: Developing supportive policies for the transition. | ⚪ | ⚪ | ⚪ | ⚪ | ⚪ |
| Monitoring and evaluation: Tracking progress and impact of the transition. | ⚪ | ⚪ | ⚪ | ⚪ | ⚪ |
| Integration with ANC services: Ensuring MMS fits into existing antenatal care workflows. | ⚪ | ⚪ | ⚪ | ⚪ | ⚪ |
| Cost-effectiveness: Evaluating financial sustainability of the transition. | ⚪ | ⚪ | ⚪ | ⚪ | ⚪ |
| Cultural acceptability: Addressing fears or misconceptions among pregnant women. | ⚪ | ⚪ | ⚪ | ⚪ | ⚪ |
| Sustainable financing. Financing for long-term MMS provision | ⚪ | ⚪ | ⚪ | ⚪ | ⚪ |

#### Wrap-up (5 minutes):

- Summarize the main points discussed and recommendations
- Ask if any important points were missed
- Thank the participants for their valuable contributions
- Share information about next steps and how their feedback will be utilized

## 2b. Focus Group Discussion Guide with Female Community Health Volunteers (FCHVs)

### Research Questions:

1. What strategies would best support the government of Nepal (local and provincial) in transitioning from IFA to MMS?

2. What operational issues may be faced by the government (local and provincial) during the transition from IFA to MMS?

#### Participants:

- Female community health volunteers that have worked for at least 5 years in their community and registered with local health center.

#### Timeframe:

- Post-enrollment > 30 days

**Introduction (5 minutes)**

- Warm welcome: Acknowledge their dedication and thank them for taking the time to share their experiences.
- Explain the purpose: Briefly explain the research is about understanding their thoughts on switching from IFA to MMS and how they can support it.
- Confidentiality: Assure them of anonymity and confidentiality.
- Obtain written informed consent.

### Topic 1: Understanding ****Women’s Experiences with MMS (20 minutes):****

- **"Currently, some women in your area have been taking MMS for about 1-2 months. Can you share any feedback—positive or negative—you heard from pregnant women on taking MMS?”**
  - **Probe: “Have women shared any concerns or side effects with you about MMS? Please list them out one by one?”**
  - **Probe: “Have you heard any pregnant women share feedback on the pill size or swallowability?**
  - **Probe: “On pill taste?” “On pill smell?**
- **“When women share their concerns about MMS, can you tell us how you typically counsel them?”**
  - **Probe: “How do you counsel regarding perceived side effects?**
- **"What are some of the challenges you face when explaining MMS?"**

### Topic 2: Support for Counselling (15 minutes):

**Ask about their training preferences and resource needs.**

- "What kind of training would help you feel confident explaining MMS to pregnant women in your community?"
  - Probe: “How frequently do you suggest we offer refresher training?”
- "Do you prefer training materials, videos, or group discussions?"
  - Probe: "What materials or tools would be most helpful for you in explaining MMS to pregnant women? For example, T

### Topic 3: Interactive Activities Free List, Ranking on MMS Information and Support (20 minutes)

#### Free List: Ask FCHVs to list everything they think they might need

- "On the piece of paper provided, please brainstorm and list all resources and tools/job aids you think you might need to support the transition to MMS and explain it effectively.”

#### Ranking

- After providing a list, put a #1 on the resource you think is most important to provide, a #2 on the second most important resource, and a #3 on the third most important resource.

NOTE: Ensure the facilitator collects everyone’s paper responses and records the responses and the rankings.

#### Discussion

Look at the top 3 rankings from the FCHVs and list them on a whiteboard. Discuss this prioritization with the group.

- “Taking a look at a few of the top rankings for training and resources, what are your thoughts on why this is important?”

### Topic 4: Prioritization

Please rate your each dimension on a scale of 1-5, where 1 is "Not at all important," and 5 is "Extremely important."

| Support resources for FCHVs | 1 | 2 | 3 | 4 | 5 |
| --- | --- | --- | --- | --- | --- |
| Training and Capacity Building   - Frequency for FCHVs to confidently counsel pregnant women about MMS. | ⚪ | ⚪ | ⚪ | ⚪ | ⚪ |
| Counseling Tools and Resources   - Need for materials like pamphlets, posters, videos, or job aids to explain MMS effectively. | ⚪ | ⚪ | ⚪ | ⚪ | ⚪ |
| Community Awareness and Engagement   - Strategies to raise awareness about MMS benefits among pregnant women, families, and communities. - Approaches to address misconceptions or fears about MMS. | ⚪ | ⚪ | ⚪ | ⚪ | ⚪ |
| Supply Chain Management   - Ensuring consistent availability of MMS at community health centers - Addressing challenges like stockouts or storage issues. | ⚪ | ⚪ | ⚪ | ⚪ | ⚪ |
| Monitoring and Feedback Mechanisms   - Systems to track feedback from pregnant women regarding their experiences with MMS. - Mechanisms to monitor adherence and identify barriers to usage. | ⚪ | ⚪ | ⚪ | ⚪ | ⚪ |
| Integration with ANC Services   - Seamless incorporation of MMS distribution into existing antenatal care workflows. - Minimizing additional workload for FCHVs. | ⚪ | ⚪ | ⚪ | ⚪ | ⚪ |
| Cultural Acceptability   - Addressing concerns related to pill size, taste, smell, or other attributes that may affect acceptability. - Tailoring counseling approaches to align with local beliefs and practices. | ⚪ | ⚪ | ⚪ | ⚪ | ⚪ |

## 2c: Focus Group Discussion Guide with Antenatal Healthcare Providers

### Research Questions:

1. What strategies would best support the government of Nepal (local and provincial) in transitioning from IFA to MMS?

2. What operational issues may be faced by the government (local and provincial) during the transition from IFA to MMS?

#### Participants:

- A mix of nurses, midwives, and doctors.

#### Timeframe:

- Post-enrollment > 30 days

#### Introduction (5 minutes)

- Welcome and thank participants for their time.
- Explain the purpose: Understand their experiences and recommendations regarding:
  - Supporting pregnant women transitioning from IFA to MMS in antenatal care in the demonstration study.
  - Raising awareness and promoting the broader adoption of MMS within the community.
- Assure confidentiality and anonymity.
- Obtain written informed consent.

### Topic 1: Understanding Pregnant Women’s Experience with MMS (20 minutes)

- "When you first introduced the pregnant women to MMS and discussed the benefits of taking MMS with them, can you tell us about the questions they asked?
  - Probe: “Were there any fears, misconceptions, or concerns?"
  - Probe: "Please share with us how you addressed these questions?” Please go over each concern and discuss how you would address it.
- “Some women in your clinic have been taking MMS for about 1-2 months. Can you share any feedback from the women about MMS?”
  - Probe: “Any comments from women on the pill size, swallowability?
  - Probe: “Any comments on the smell?
  - Probe: “Any comments from women on the pill color?
- In your opinion, what messages have proven effective in addressing the anxieties or questions pregnant women have regarding MMS?

### Topic 2: Counseling Support for MMS (15 minutes)

- “At the project start, health providers were provided with a 1-day training on MMS. Please share openly if you think this 1-day training was sufficient in helping you counsel pregnant women on the benefits of MMS and helped you answer all the pregnant women’s questions on MMS. Why or why not?”
  - Probe: “If more training time is needed, please let us know what additional topic areas or practice sessions are needed (i.e., a session on how to address common concerns or a session for role-playing?”
  - Probe: “Are there any resources or job aids that would be valuable for you to confidently advise pregnant women about MMS (i.e., pamphlets, posters, videos, take-home materials)?

### Topic 3: Supporting Successful Transition (10 minutes):

- “As we discussed before, the Government of Nepal is considering transitioning from IFA to MMS as part of antenatal care. What strategies would be most effective in supporting this transition at both the local and provincial levels?
  - Probe: “What types of community engagement and awareness campaigns can be used to share information about the advantages of the transition to MMS (i.e. community dramas, social media videos, radio programs?)
- Are there any specific challenges or considerations unique to certain areas or communities in Nepal that must be addressed during the transition?

### Topic 4: Anticipating Operational Issues (15 minutes):

- “While transitioning from IFA to MMS, do you anticipate the government may face logistical and operational issues at both the local and provincial levels?” Some issues may be in health worker training, stockout of MMS, storage and distribution, and increased workload.

Activity: On the sheet of paper provided, brainstorm and list a few operational issues you anticipate the provincial and district governments could face.

After providing a list, put a #1 on the issue that you think is most important to address; put a #2 on the second most important issue, and a #3 on the third most important issue.

NOTE: Ensure the facilitator collects everyone’s paper responses and records the responses and the rankings.

- “Please share some of your top concerns. What strategies can be used to address these challenges?”
- “Are there any perceived challenges around health worker capacity to support the transition?” What are some solutions?
- “Are there any perceived challenges with increased workload or changes in service delivery associated with the transition?” What are some solutions?
- “Are there any perceived challenges with MMS storage in the health facility?” What are some solutions?
- “Are there any perceived challenges with MMS stockouts?” What are some solutions?

### Topic 5: Prioritization

Please rate your priority on a scale of 1-5, where 1 is "Not at all important," and 5 is "Extremely important."

| Key health system dimensions | 1 | 2 | 3 | 4 | 5 |
| --- | --- | --- | --- | --- | --- |
| Health worker training: Training adequacy and capacity building for healthcare providers. | ⚪ | ⚪ | ⚪ | ⚪ | ⚪ |
| Supply chain management: Addressing stockouts, storage, and distribution challenges | ⚪ | ⚪ | ⚪ | ⚪ | ⚪ |
| Community awareness: Raising public knowledge about MMS benefits. | ⚪ | ⚪ | ⚪ | ⚪ | ⚪ |
| Policy framework: Developing supportive policies for the transition. | ⚪ | ⚪ | ⚪ | ⚪ | ⚪ |
| Monitoring and evaluation: Tracking progress and impact of the transition. | ⚪ | ⚪ | ⚪ | ⚪ | ⚪ |
| Integration with ANC services: Ensuring MMS fits into existing antenatal care workflows. | ⚪ | ⚪ | ⚪ | ⚪ | ⚪ |
| Cost-effectiveness: Evaluating financial sustainability of the transition. | ⚪ | ⚪ | ⚪ | ⚪ | ⚪ |
| Cultural acceptability: Addressing fears or misconceptions among pregnant women. | ⚪ | ⚪ | ⚪ | ⚪ | ⚪ |

#### Wrap-up (5 minutes)

- Summarize key points and recommendations discussed. Ask if important points were missed.
- Thank participants for their valuable contributions.
- Share information about next steps and how their feedback will be utilized.

Additional Tips:

- Encourage open and honest dialogue but be mindful of cultural sensitivities.
- Consider using case studies or scenarios to spark discussion and explore practical approaches.
- Adapt the discussion flow based on the group's responses and interests.
- Consider the specific context and challenges of the healthcare facilities your participants work in.
- Provide visual aids or resources to facilitate understanding and encourage further engagement.
