# Supplementary material for: Feasibility and Readiness for Scaling‐Up Multiple Micronutrient Supplements in Nepal: A Qualitative Study Using the Expandnet Scaling‐Up Framework
Source: Matern Child Nutr. 2026 Jul 17;22(3):e70231. doi: 10.1111/mcn.70231 (PMC13377524; doi:10.1111/mcn.70231)

Figure 1. Illustration of the coding tree used for the analysis based on ExpandNet's framework for scaling-up

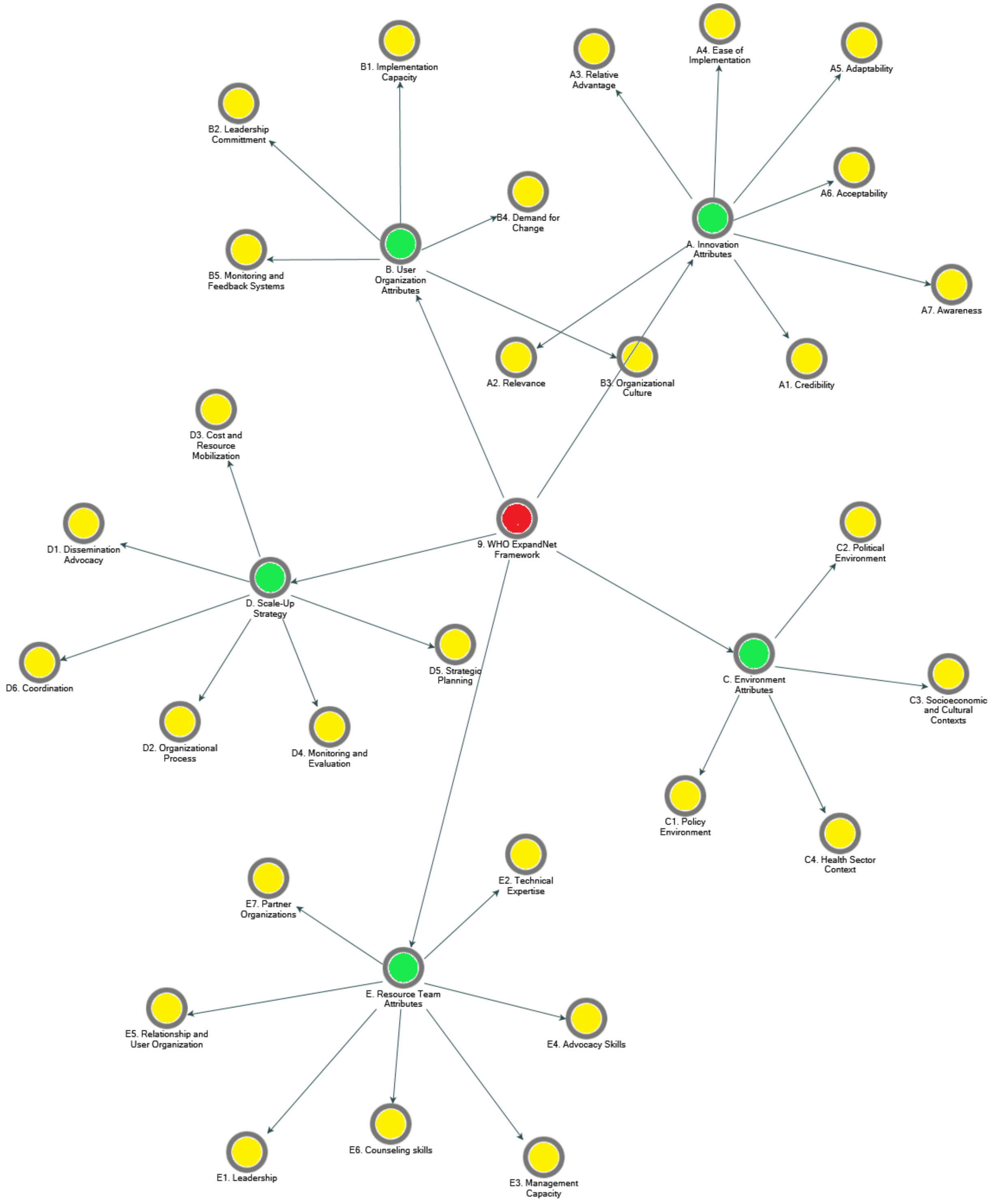

Supplement: Supplementary file 3 — Supporting File 3 [file MCN-22-e70231-s002.pdf]
