# Supplementary material for: Feasibility and Readiness for Scaling‐Up Multiple Micronutrient Supplements in Nepal: A Qualitative Study Using the Expandnet Scaling‐Up Framework
Source: Matern Child Nutr. 2026 Jul 17;22(3):e70231. doi: 10.1111/mcn.70231 (PMC13377524; doi:10.1111/mcn.70231)
Supplement: Supplementary file 4 — Supporting File 4 [file MCN-22-e70231-s003.docx]

# **Supplementary File 4: Codebooks with Antenatal Care Providers, Female Community Health Volunteerss, and Policymakers**

## 4a. Codebook for Focus Group Discussions with ANC Healthcare Providers using WHO ExpandNet Framework

### Overview of the Codebook

This codebook organizes qualitative data into five thematic domains aligned with the WHO ExpandNet framework components. Each domain contains parent-child codes derived from the FGD guide and research questions, capturing insights on implementation strategies, barriers, facilitators, and sustainability factors related to the IFA to MMS transition.

### WHO ExpandNet Framework Alignment

The codebook maps to the 5 core components of the WHO ExpandNet framework:

1. Innovation (MMS as the targeted intervention)
2. Resource Team (healthcare providers implementing MMS)
3. User Organizations (health facilities and systems adopting MMS)
4. External Environmental Context (sociopolitical factors, community needs)
5. Scaling-up Strategies (approaches for transitioning from IFA to MMS)

### Implementation Guidelines

- **Hierarchical Coding**: Create parent codes based on ExpandNet components and nest FGD themes as child codes
- **Deductive Coding**: Apply pre-defined codes to transcripts using the "When to Use" criteria
- **Inductive Coding**: Add emergent themes as new codes, then map them to the framework
- **Coding Process**: Begin with broad codes, then move to more specific subcodes while maintaining context

### Table 1: Innovation Attributes (MMS Implementation)

| Code | Definition | When to Use | Example |
| --- | --- | --- | --- |
| A_1. MMS Characteristics | Physical and perceived attributes of MMS as a healthcare intervention | When participants discuss features or practical aspects of MMS | "The pill size is smaller compared to IFA" |
| A_1.1. Physical attributes | Physical properties of MMS pills that affect acceptability | When discussing pill size, color, smell, swallowability | "Women reported disliking the smell of the pills" |
| A_1.2. Perceived effectiveness | Perceptions of MMS benefits compared to IFA | When discussing perceived health benefits or advantages | "Providers noted that women reported less nausea with MMS" |
| A_1.3. Side effects | Reported or perceived side effects of MMS | When discussing adverse effects or concerns about side effects | "Some women experienced mild gastrointestinal discomfort" |
| B_2. Women's Responses to MMS | Pregnant women's experiences, feedback, and concerns | When discussing how women have responded to MMS introduction | "After taking MMS for a month, women provided positive feedback" |
| B_2.1. Initial concerns | Questions or concerns raised when first introduced to MMS | When discussing initial reactions, fears, or misconceptions | "Women initially worried about multiple nutrients causing large babies" |
| B_2.2. Ongoing feedback | Feedback received after using MMS for some time | When discussing experiences after 1-2 months of use | "Women taking MMS for two months reported fewer side effects" |
| B_2.3. Misconceptions | Common misconceptions about MMS | When discussing incorrect beliefs about MMS | "Some women believed MMS would cause birth defects" |
| C_3. Effective Messaging | Communication strategies proven effective for MMS adoption | When discussing successful approaches to explaining MMS | "Visual aids showing different nutrients helped women understand benefits" |
| C_3.1. Addressing anxieties | Approaches to address women's concerns | When discussing how providers alleviate anxieties | "Explaining MMS safety record helped reduce fears" |
| D_3.2. Explaining benefits | Strategies to explain MMS benefits effectively | When discussing explanation of advantages | "Relating nutrients to baby's development was effective" |

### Table 2: Resource Team (Healthcare Provider Capacity)

| Code | Definition | When to Use | Example |
| --- | --- | --- | --- |
| E_1. Training Adequacy | Sufficiency and effectiveness of training provided | When discussing whether MMS training was adequate | "The one-day training was insufficient to cover counseling needs" |
| E_1.1. Training content needs | Specific areas requiring more attention in training | When discussing gaps in training content | "We needed more information on managing side effects" |
| E_1.2. Training format | Comments on structure, duration, or delivery method | When discussing aspects of training delivery | "Role-playing would be more effective than lectures" |
| E_1.3. Confidence in counseling | Providers' confidence in their ability to counsel | When discussing comfort level with providing information | "I still felt unsure about addressing certain concerns" |
| F_2. Support Materials | Resources that support providers in counseling | When discussing job aids or reference materials | "A pamphlet would reinforce messages we provide" |
| F_2.1. Job aids | Tools helping providers deliver consistent information | When discussing materials used during consultations | "A counseling flipchart would help cover all points" |
| F_2.2. Take-home materials | Materials given to pregnant women as reinforcement | When discussing printed materials for women | "Women wanted written information to share with family" |
| F_2.3. Visual aids | Visual materials explaining MMS benefits or usage | When discussing pictures, charts, or visual tools | "A nutrient comparison chart helps explain differences" |
| G_3. Provider Attitudes | Healthcare providers' perspectives toward MMS | When discussing how providers feel about the transition | "Providers were enthusiastic about offering comprehensive supplements" |
| G_3.1. Perceived value | Providers' perception of MMS importance | When discussing whether providers see MMS as beneficial | "All providers agreed MMS offers significant advantages" |
| G_3.2. Implementation challenges | Provider-reported difficulties in implementation | When discussing challenges in introducing MMS | "Finding time for in-depth counseling is difficult" |

### Table 3: User Organizations (Health System Factors)

| Code | Definition | When to Use | Example |
| --- | --- | --- | --- |
| H_1. Logistical Considerations | Practical aspects of MMS implementation in facilities | When discussing supply chain or distribution issues | "Storage space for MMS is limited in smaller health posts" |
| H_1.1. Storage | Issues related to storing MMS at health facilities | When discussing storage conditions or challenges | "MMS requires cool, dry storage which is challenging" |
| H_1.2. Distribution | Process of distributing MMS to pregnant women | When discussing how MMS reaches women | "Monthly distribution requires women to travel frequently" |
| H_1.3. Stock management | Management of MMS supplies and stock levels | When discussing inventory management or stockouts | "We need better systems to track stock levels" |
| I_2. Workload Implications | Impact of MMS transition on providers' workload | When discussing changes in responsibilities | "Additional counseling requires more time per visit" |
| I_2.1. Time constraints | Issues related to time needed for MMS counseling | When discussing time pressures | "Busy clinics lack time to explain MMS properly" |
| I_2.2. Integration with services | How MMS counseling fits with other services | When discussing integration into routine care | "MMS counseling can be incorporated into standard packages" |
| I_2.3. Documentation | Record-keeping and reporting related to MMS | When discussing paperwork or reporting | "Additional forms add to the workload" |
| J_3. Health Facility Capacity | Capability of facilities to support MMS implementation | When discussing facility readiness | "Smaller health posts lack staff capacity for transition" |
| J_3.1. Infrastructure | Physical infrastructure needed for implementation | When discussing facility conditions | "Private counseling spaces are needed for addressing concerns" |
| J_3.2. Human resources | Staffing considerations for implementation | When discussing staffing levels or roles | "Additional staff may be needed during transition" |

### T**able 4: External Environmental Context (Community and Policy Factors)**

| Code | Definition | When to Use | Example |
| --- | --- | --- | --- |
| K_1. Community Awareness | Level of community knowledge about MMS | When discussing community understanding | "Communities have little prior knowledge about MMS" |
| K_1.1. Engagement strategies | Approaches for engaging communities | When discussing awareness-raising methods | "Community radio could help spread information" |
| K_1.2. Information channels | Communication channels for reaching communities | When discussing information dissemination | "FCHVs are effective messengers for nutrition information" |
| K_1.3. Cultural considerations | Cultural factors influencing MMS acceptance | When discussing cultural beliefs affecting uptake | "Mothers-in-law often make decisions about supplements" |
| L_2. Geographic Challenges | Challenges related to geography or location | When discussing geographical access issues | "Women in remote areas face difficulties accessing facilities" |
| L_2.1. Access to services | Accessibility of health services for pregnant women | When discussing physical access to healthcare | "Monsoon season cuts off some communities" |
| L_2.2. Regional variations | Differences between regions in implementation needs | When discussing location-specific needs | "Terai and hill regions require different strategies" |
| M_3. Policy Environment | Policy factors influencing MMS implementation | When discussing policies or government positions | "Current guidelines still recommend IFA as standard" |
| M_3.1. Policy alignment | Alignment of MMS with existing health policies | When discussing how MMS fits with policies | "MMS aligns with nutrition policy's focus on stunting" |
| M_3.2. Government prioritization | Level of government priority for maternal nutrition | When discussing government commitment | "Provincial governments vary in prioritization" |
| M_3.3. Resource allocation | Allocation of resources for maternal nutrition | When discussing funding or resource issues | "Budget allocations for supplements are insufficient" |

### Table 5: Scaling-up Strategies (Transition Approaches)

| Code | Definition | When to Use | Example |
| --- | --- | --- | --- |
| N_1. Transition Strategies | Approaches for managing IFA to MMS transition | When discussing implementation approaches | "A phased approach starting with high-volume facilities could work" |
| N_1.1. Phased implementation | Implementing MMS in phases or stages | When discussing gradual implementation | "Begin with district hospitals, then expand to health posts" |
| N_1.2. Communication planning | Planning for communicating the transition | When discussing explaining the transition | "Clear communication needed to explain the change" |
| N_1.3. Managing parallel systems | Managing both IFA and MMS during transition | When discussing coexistence of supplements | "Guidance needed on existing IFA stocks during transition" |
| O_2. Operational Challenges | Challenges in operationalizing the transition | When discussing anticipated operational issues | "Stockouts may occur if supply chains aren't strengthened" |
| O_2.1. Supply chain concerns | Issues related to MMS procurement and supply | When discussing supply challenges | "International procurement may cause availability delays" |
| O_2.2. Training scale-up | Scaling up training for healthcare providers | When discussing provider training approaches | "Cascade training can reach remote providers efficiently" |
| O_2.3. Monitoring systems | Systems for monitoring transition and outcomes | When discussing monitoring and evaluation | "Simple tools needed to track distribution and adherence" |
| P_3. Sustainability Planning | Approaches to ensure long-term sustainability | When discussing how to make MMS sustainable | "Local production could reduce costs and ensure sustainability" |
| P_3.1. Resource mobilization | Strategies for securing resources for MMS | When discussing funding approaches | "Donors could support initial costs while government builds capacity" |
| P_3.2. Integration into systems | Integrating MMS into routine health systems | When discussing normalization of MMS | "MMS needs integration into essential medicines list" |
| P_3.3. Capacity building | Building long-term capacity for implementation | When discussing capability development | "Local capacity for production and quality control is essential" |
| Q_4. Stakeholder Engagement | Engaging with various stakeholders in transition | When discussing involvement of different actors | "Private providers need inclusion in transition planning" |
| Q_4.1. Coordination mechanisms | Mechanisms for coordinating among stakeholders | When discussing coordination approaches | "Technical working group could coordinate across ministries" |
| Q_4.2. Role definitions | Defining roles and responsibilities | When discussing responsibility allocation | "Clear division needed between federal and provincial levels" |
| Q_4.3. Advocacy approaches | Approaches for advocating for MMS adoption | When discussing advocacy strategies | "Evidence from demonstration study useful for advocacy" |

## 4b. Codebook for Female Community Health Volunteer FGD using WHO ExpandNet Framework

**Codebook for NVivo 14: Focus Group Discussions with FCHVs on IFA to MMS Transition in Nepal**

**Study Information**

**Research Questions**:

1. What strategies would best support the government of Nepal (local and provincial) in transitioning from IFA to MMS?
2. What operational issues may be faced by the government (local and provincial) during the transition from IFA to MMS?

**Participants**: Female community health volunteers with at least 5 years of experience in their community and registered with local health center.

Overview of the Codebook
This codebook provides a structured framework for analyzing FGDs with FCHVs in Nepal regarding the transition from IFA to MMS. It aligns qualitative themes with the **WHO ExpandNet Framework** to ensure systematic evaluation of scalability factors while capturing local operational realities. Below is a guide to its structure, alignment, and application:

### 1. Codebook Structure

The codebook organizes data into **8 thematic tables**, each containing **parent-child codes** derived from the FGD guide and research questions:

| Table | Focus | Key Codes |
| --- | --- | --- |
| 1. Transition Strategies | Government support strategies for transitioning to MMS | Focuses on approaches and methods for shifting from IFA to MMS, directly aligned with scale-up plans. |
| 2. Operational Issues | Practical challenges during implementation | Addresses facility-level, system, and workflow barriers-core to health system adoption and readiness. |
| 3. Women’s Experiences | Pregnant women’s feedback on MMS | Captures end-user perceptions and experiences with MMS, reflecting attributes of the innovation. |
| 4. FCHV Counseling | Techniques for communicating about MMS | Encompasses FCHV skills, training, and counseling-core aspects of the implementing resource team. |
| 5. Support Resources | Tools/training needed by FCHVs | Relates to needs for materials, incentives, and support for FCHVs as implementers.. |
| 6. Delivery Setting | Context of MMS distribution | Pertains to where and how services are delivered-organizational and facility-level considerations. |
| 7. Acceptability | Cultural and product-related factors affecting MMS adoption | Pill taste/size, social influence, perceived value. |
| 8. Reach | Coverage and equity issues | Focuses on equity, coverage, and reaching marginalized populations-key to scale-up and adaptation. |

**WHO ExpandNet Framework Alignment**

The codebook maps themes to **5 core components** of the WHO framework to evaluate scalability:

| WHO Component | Codebook Tables Aligned | Example Codes |
| --- | --- | --- |
| Innovation Attributes |  | Captures end-user perceptions and experiences with MMS, reflecting attributes of the innovation. |
| User Organization Attributes |  | Addresses facility-level, system, and workflow barriers-core to health system adoption and readiness. |
| Environment Attributes |  | Ecological/physical context or sociocultural context; policy context |
| Scale-Up Strategy |  | Focuses on equity, coverage, and reaching marginalized populations-key to scale-up and adaptation. |
| Resource Team Attributes |  | Relates to needs for materials, incentives, and support for FCHVs as implementers. |

### Implementation Guidelines In NVivo 14/15

- **Hierarchical Coding:**
  - Create parent codes or WHO ExpandNet components (e.g., *Innovation Attributes*).
  - Nest codebook themes (e.g., *Women’s Experiences*) as child codes.
- **Deductive Coding:** Apply pre-defined codes to transcripts using the codebook’s "When to Use" criteria.
- **Inductive Coding:** Add emergent themes (e.g., "community taboos") as new codes, then map them to frameworks.

**Table 1: Transition Strategies**

| **Code** | **Description** | **When to Use** | **When Not to Use** | **WHO ExpandNet Framework Strategy** |
| --- | --- | --- | --- | --- |
| **1. Transition Strategies** | References to approaches, mechanisms, or actions that would support the government of Nepal in transitioning from IFA to MMS distribution. | Apply this code to content discussing specific methods, plans, or frameworks for facilitating the transition process, including policy changes, resources needed, and implementation approaches. | Do not use for general discussions about MMS that don't specifically relate to the transition process or government support. | **Scale-Up Strategy** (Strategic planning, advocacy, resource mobilization) |
| **1.1 Policy Integration** | References to incorporating MMS into existing health policies, guidelines, protocols, or national strategies. | When participants discuss how MMS should be included in official documents, policies, or national health programs. | For discussions about general implementation without specific mention of policy documents or formal guidelines. | **Scale-Up Strategy** (Strategic planning, advocacy, resource mobilization) |
| **1.2 Training and Capacity Building** | Mentions of training needs, approaches, or programs to equip health workers and FCHVs with knowledge and skills related to MMS. | When discussing specific training content, frequency, methodology, or resources needed for education about MMS. | For general mentions of knowledge gaps without specific training recommendations. | **Resource Team** (Encompasses FCHV skills, training, and counseling-core aspects of the implementing resource team). |
| **1.2a Training modality** | References to preferred or suggested methods for delivering MMS training (e.g., in-person, online, blended, peer-to-peer). | When participants specify the format, platform, or style of training delivery for MMS. | When training is mentioned without specifying modality or delivery method. | **Resource Team**  **(**Training approach, capacity building) |
| **1.2a.1 Request for physical sample of MMS to show women** | Requests for physical MMS samples to be used during training or counseling to improve understanding and acceptance. | When participants specifically request actual MMS tablets or packaging for demonstration purposes. | When discussing training without mention of physical samples. | **Resource Team**  **(Job aids, demonstration tools)** |
| **1.2b Training Duration** | References to the length or scheduling of MMS training sessions. | When participants discuss how long training should be, or preferred timing/frequency. | When training is mentioned without reference to duration or scheduling. | **Resource Team**  (Training logistics, planning) |
| **1.3 Resource Allocation** | References to financial, human, or material resources needed for successful transition. | When participants discuss budgeting, funding requirements, staffing needs, or material resources required. | For general discussions about challenges without specific resource implications. | **Scale-Up Strategy** (Strategic planning, advocacy, resource mobilization) |
| **1.3a. Resources_Incentives** | When FCHVs say that incentives are needed to motivate performance and ensure a successful transition | When participants specifically mention incentives (financial or non-financial) for FCHVs or other stakeholders. | When incentives are not mentioned or are discussed in a general sense unrelated to the transition. | **Scale-Up Strategy** > resource mobilization |
| **1.3b. Resources_mass media** | When mass media is noted as an important IEC strategy and for training | When participants discuss using radio, TV, social media, or other mass media for awareness or training. | When media is not mentioned, or only interpersonal communication is discussed. | **Scale-Up Strategy** > D1. Dissemination Advocacy |
| **1.4 Phased Implementation** | Suggestions for gradual or staged implementation of the transition from IFA to MMS. | When participants discuss rollout schedules, pilot programs, or geographical prioritization for implementation. | For discussions about immediate, full-scale implementation. | **Scale-Up Strategy** (Guided expansion, adaptive implementation) |
| **1.5. Awareness raising needs** | References to needing broader awareness-raising activities among key stakeholders. | When participants discuss the need for awareness strategies. | When participants discuss specific | **Scale-Up Strategy** (Guided expansion, adaptive implementation) |

### Table 2: Operational Issues

| **Code** | **Description** | **When to Use** | **When Not to Use** | **WHO ExpandNet Framework Strategy** |
| --- | --- | --- | --- | --- |
| **2. Operational Issues** | References to practical challenges, barriers, or considerations in implementing the transition from IFA to MMS. | Apply this code to content discussing logistics, workflow changes, supply chain considerations, and other practical implementation factors. | Do not use for content focused on policy-level strategy or theoretical benefits of MMS without operational implications. | **Scale-Up Strategy** (Guided expansion, adaptive implementation) |
| **2.1 Workload Impact** | References to how the transition affects FCHVs' and health workers' responsibilities, time, or work burden. | When participants discuss changes to their daily activities, additional tasks, or time constraints related to MMS transition. | For discussions about general challenges not specifically related to workload or time requirements. | **User Organization Attributes** (Organizational capacity, staff burden) |
| **2.2 Supply Chain Management** | References to procurement, storage, distribution, or inventory management of MMS. | When participants discuss how MMS will be ordered, stored, transported, tracked, or distributed. | For discussions about MMS characteristics or benefits without logistical considerations. | **Scale-Up Strategy** (Logistics, sustainability planning) |
| **2.3 Integration with Existing Services** | References to how MMS distribution will fit within current antenatal care services or health system functions. | When participants discuss coordination with other services, workflow adjustments, or system integration considerations. | For discussions about completely new systems or processes without reference to existing structures. | **Scale-Up Strategy** (Guided expansion, adaptive implementation) |
| **2.4 Documentation and Reporting** | References to record-keeping, monitoring, reporting requirements, or data collection related to MMS. | When participants discuss forms, registers, digital tools, or reporting processes needed for MMS tracking. | For general discussion about monitoring without specific documentation mechanisms. | **User Organization Attributes** (Organizational capacity, staff burden) |

### Table 3: Women's Experiences with MMS

| **Code** | **Description** | **When to Use** | **When Not to Use** | **WHO ExpandNet Framework Strategy** |
| --- | --- | --- | --- | --- |
| **3. Women's Experiences with MMS** | References to pregnant women's feedback, experiences, perceptions, or reactions to MMS. | Apply this code to content discussing reported benefits, side effects, concerns, or perspectives from pregnant women who have taken MMS. | Do not use for FCHVs' personal opinions about MMS unless they are specifically reporting what women have shared with them. | **Innovation Attributes** (Perceived value, relevance to users) |
| **3.1 Reported Benefits** | References to positive outcomes or effects that women have attributed to MMS use. | When participants report women's statements about health improvements, energy levels, or other positive experiences with MMS. | For theoretical benefits that women haven't personally reported experiencing. | **Innovation Attributes** (Captures end-user perceptions and experiences with MMS, reflecting attributes of the innovation.) |
| **3.2 Side Effects and Concerns** | References to negative experiences, discomfort, or worries reported by women taking MMS. | When participants discuss specific side effects women have reported or concerns women have raised about taking MMS. | For theoretical side effects that FCHVs believe might occur but haven't been reported by women. | **Innovation Attributes** (Captures end-user perceptions and experiences with MMS, reflecting attributes of the innovation.) |
| **3.3 Physical Characteristics Feedback** | References to women's comments about pill size, taste, smell, color, packaging, or other physical attributes of MMS. | When participants relay women's specific feedback about the sensory or physical properties of MMS tablets. | For general discussions about pill characteristics without specific feedback from women. | **Innovation Attributes** (Design adaptability) |
| **3.4 Adherence Patterns** | References to women's consistency in taking MMS as prescribed, including missed doses or discontinuation. | When participants discuss patterns they've observed or women have reported regarding regular or irregular consumption of MMS. | For theoretical discussions about adherence factors without specific examples. | **Innovation Attributes** (Captures end-user perceptions and experiences with MMS, reflecting attributes of the innovation.) |
| **3.5 Cultural Beliefs and Practices** | References to cultural factors, traditions, or beliefs that influence MMS acceptance. | When participants discuss how local cultural perspectives affect women's willingness to take MMS. | For general discussions about barriers without cultural context. | **Environment Attributes** (Sociocultural context |
| **3.6 Perceived Value and Benefits** | References to how women's understanding of MMS benefits affects their acceptance. | When participants discuss how women's perceptions of MMS value influence their willingness to take it. | For factual discussions about scientific benefits without perception component. | **Innovation Attributes  (**Relative advantage, observability) |
| **3.6a. Other perceived side effects** | References to other side effects that women mention that are either neutral in sentiment (not negative ) |  |  | **Innovation Attributes** |
| **3.7 Social Influence** | References to how family members, peers, or community leaders influence MMS acceptance. | When participants discuss the role of social networks or authority figures in MMS decisions. | For individual preference factors without social component. | **Environmental Attributes**  (Sociocultural context) |
| **3.8 Inadequate knowledge or Misconceptions about MMS** | References to knowledge of women regarding MMS or any misconceptions of women or lack of knowledge regarding MMS | When women express uncertainty or incorrect beliefs about MMS. | Factual knowledge without reference to women's understanding. | **User Organization/Environment**  **(**Capacity to adopt, information needs) |
| **3.9 Considerations for Guideline revisions** | When there are indications that future guidelines on MMS or ANC should be revised or updated | When feedback suggests changes to current protocols or recommendations. | Routine discussions not related to policy or guideline change. | **Scale-Up Strategy**  **(**Policy adaptation, system readiness) |
| **3.10 Perceived birth outcomes** | When women discuss their perceived birth outcomes based on taking MMS | When women discuss birth outcomes they associate with MMS. | Clinical outcomes not linked to women's perceptions. | **Innovation Attributes  (**Observed effects, user-reported outcomes) |
| **3.11 Questions about how to take MMS** | Women's questions or uncertainties about MMS regimen or instructions. | When women ask about timing, dosage, or administration of MMS. | Instructional content not reflecting women's questions. | **User Organization/Environment** (Training needs, clarity of use) |

### Table 4: FCHV Counseling Practices

| **Code** | **Description** | **When to Use** | **When Not to Use** | **WHO ExpandNet Framework Strategy** |
| --- | --- | --- | --- | --- |
| **4. FCHV Counseling Practices** | References to how FCHVs communicate with, educate, or support pregnant women regarding MMS. | Apply this code to content discussing specific techniques, approaches, challenges, or experiences related to providing information or addressing concerns about MMS. | Do not use for general discussions about MMS information that don't specifically relate to FCHV-woman interactions. | **Resource Team** (Encompasses FCHV skills, training, and counseling-core aspects of the implementing resource team.) |
| **4.1 Counseling Strategies** | References to specific methods, language, or approaches FCHVs use when explaining MMS to women. | When participants describe their communication techniques, explanations, or persuasion tactics when discussing MMS with women. | For general educational needs without specific counseling approaches. | **Resource Team Attributes** (Training quality, communication skills) |
| **4.2 Addressing Misconceptions** | References to how FCHVs respond to or correct misinformation, myths, or misunderstandings about MMS. | When participants describe specific ways they address women's incorrect beliefs or fears about MMS. | For general discussions about misconceptions without specific correction strategies. | **Innovation Attributes** (Cultural compatibility) |
| **4.3 Counseling Challenges** | References to difficulties FCHVs face when explaining MMS or convincing women to take it. | When participants describe specific barriers or problems they encounter during counseling sessions about MMS. | For general challenges not specifically related to communicating about MMS. | **Resource Team Attributes** (Training quality, communication skills) |
| **4.4 Family Involvement** | References to engaging family members, especially husbands or mothers-in-law, in MMS counseling or decision-making. | When participants discuss how they include or address family influence in MMS acceptance and adherence. | For general community engagement without specific family component. | **Environment Scale-Up Strategy** (Sociocultural context) |
| **4.4a Family impression regarding MMS** | When the family members give feedback regarding  MMS | When family members respond positively or negatively to MMS, as reported by FCHVs. | For FCHV or women's impressions not involving family. | **Environment Attributes**  (family and community context) |
| **4.5. FCHVs inadequate knowledge or misconception** | When FCHVs say something about MMS that is “incorrect” or indicates a misunderstanding | When FCHVs express or demonstrate misconceptions about MMS facts. | For correct knowledge or general lack of information not specific to misconceptions. | **Resource Team Attributes**  (training needs, knowledge gaps) |
| **4.6 FCHVs’ Knowledge and Understandings** | FCHVs’ knowledge and understandings regarding MMS | When FCHVs accurately discuss MMS, its benefits, or protocols. | For knowledge unrelated to MMS or not from FCHVs. | **Resource Team Attributes**  **(**capacity, knowledge base) |

### Table 5: Support Resources Needed

| **Code** | **Description** | **When to Use** | **When Not to Use** | **WHO ExpandNet Framework Strategy** |
| --- | --- | --- | --- | --- |
| **5. Support Resources Needed** | References to materials, tools, training, or support systems FCHVs desire to effectively promote MMS. | Apply this code to content discussing specific resources requested by FCHVs or gaps in current support. | Do not use for general challenges without specific resource requests or solutions. | **Resource Team Attributes** (Technical support, training materials)  **User Organization Attributes** (Capacity-building needs) |
| **5.1a_Flipcharts** | equests for flipcharts as visual aids for counseling. | When FCHVs specifically mention flipcharts as a need. | When flipcharts are not mentioned or are discussed in general terms. | Resource Team Attributes (job aids, training tools) |
| **5.1b_Counseling cards** | Requests for counseling cards to guide or standardize messaging. | When FCHVs request counseling cards or reference their use. | When counseling cards are not specifically mentioned. | Resource Team Attributes (job aids, training tools) |
| **5.1c_Checklists** | Requests for checklists to ensure consistent delivery of key messages. | When FCHVs mention checklists for tracking or quality assurance. | When checklists are not specifically mentioned. | Resource Team Attributes (job aids, quality assurance tools) |
| **5.1d_Videos** | Requests for video materials for training or community education. | When FCHVs request videos as a resource for themselves or for group education. | When videos are not specifically mentioned. | Resource Team Attributes (training tools, visual aids) |
| **5.1e_Printed materials** | Requests for pamphlets, brochures, or other printed information. | When FCHVs request or reference printed materials. | When printed materials are not specifically mentioned. | Resource Team Attributes (information dissemination tools) |
| **5.1f_Demonstration materials** | Requests for tangible items to demonstrate MMS use or benefits. | When FCHVs mention items for demonstration (e.g., sample tablets, props). | When demonstration is discussed without reference to specific materials. | Resource Team Attributes (demonstration tools) |
| **5.1g_Bags for carrying materials and jobs** | Requests for bags or containers to transport job aids and materials. | When FCHVs mention the need for bags to carry materials. | When bags are not specifically mentioned. | Resource Team Attributes (logistical support) |
| **5.1 Job Aids and Materials** | References to physical or digital tools FCHVs want to support their counseling on MMS. | When participants describe specific materials like flipcharts, pamphlets, videos, or other tools they need. | For general statements about needing support without specific material requests. | **Resource Team Attributes** (Technical support, training materials) |
| **5.2 Supervision and Feedback** | References to ongoing guidance, mentoring, or performance feedback desired by FCHVs. | When participants discuss the type of supervision or feedback that would help them improve their MMS counseling. | For general discussions about program oversight without specific supervision needs. | **Resource Team Attributes** (Technical support, training materials) |
| **5.3 Peer Support Systems** | References to FCHV networks, sharing sessions, or collaborative learning approaches. | When participants discuss how they could learn from or support each other in MMS promotion. | For discussions about formal training without peer component. | **Resource Team Attributes** (Technical support, training materials)  **User Organization Attributes** (Capacity-building needs) |

### Table 6: Delivery Setting

| **Code** | **Description** | **When to Use** | **When Not to Use** | **WHO ExpandNet Framework Strategy** |
| --- | --- | --- | --- | --- |
| **6. Delivery Setting** | References to the context, environment, or system in which MMS is distributed or administered. | Apply this code to content discussing health system infrastructure, distribution points, or service delivery mechanisms related to MMS. | Do not use for discussions about MMS characteristics or benefits unrelated to delivery context. | **Environment Attributes** (Sociopolitical;’ geographic context) |
| **6.1 Distribution Channels** | References to where and how MMS is or should be physically distributed to pregnant women. | When participants discuss specific locations, events, or mechanisms for getting MMS to women. | For discussions about counseling that don't involve actual distribution. | **Scale-Up Strategy** (Dissemination pathways) |
| **6.2 Health System Integration** | References to how MMS distribution fits within existing health services, particularly antenatal care. | When participants discuss coordination with other services or programs within the health system. | For standalone discussions about MMS without reference to the broader health system. | **Scale-Up Strategy** (Dissemination pathways) |
| **6.3 Geographical Considerations** | References to location-specific factors affecting MMS delivery, particularly in remote or challenging areas. | When participants discuss how geography, distance, terrain, or regional differences affect MMS distribution. | For general challenges not specifically related to geographical factors. | **Environment Attributes** (Geographic context) |
| **6.4 Service Provider Roles** | References to who is responsible for different aspects of MMS distribution and counseling. | When participants discuss the division of responsibilities between FCHVs, health workers, and others. | For general service descriptions without specific role definitions. | **Resource Team Attributes** (Training quality, communication skills) |

### Table 7: Reach

| **Code** | **Description** | **When to Use** | **When Not to Use** | **WHO ExpandNet Framework Strategy** |
| --- | --- | --- | --- | --- |
| **7. Reach** | References to the extent to which MMS distribution reaches intended beneficiaries across different population segments. | Apply this code to content discussing coverage, access, equity, or barriers to reaching all eligible pregnant women. | Do not use for content focused on acceptance without reference to population coverage. | **Environment Attributes** (Equity, geographic accessibility) |
| **7.1 Coverage Patterns** | References to which populations or subgroups are receiving or not receiving MMS. | When participants discuss patterns of who is accessing MMS and who is being missed. | For discussions about individual experiences without population-level implications. | **Monitoring & Evaluation** (Equity analysis) |
| **7.2 Access Barriers** | References to factors preventing women from obtaining MMS, including distance, cost, or availability. | When participants discuss specific obstacles that prevent women from accessing MMS. | For acceptance barriers after MMS is already obtained. | **Environment Attributes** (Equity context; socio-cultural and socio-political context) |
| **7.3 Equity Considerations** | References to disparities in MMS access or uptake among different socioeconomic, ethnic, or geographical groups. | When participants discuss how certain groups may be disadvantaged in accessing MMS. | For general access issues without the equity dimension. | **Environment Attributes** (Equity context; socio-cultural and socio-political context) |
| **7.4 Outreach Strategies** | References to approaches for reaching underserved populations or improving coverage. | When participants discuss specific methods to extend MMS distribution to hard-to-reach women. | For general service improvements without a specific outreach focus. | **Scale-Up Strategy** (Advocacy, communication, dissemination) |

### Table 8: FCHVs_No Orientation MMS

| **Code** | **Description** | **When to Use** | **When Not to Use** | **WHO ExpandNet Framework Strategy** |
| --- | --- | --- | --- | --- |
| 1. FCHVs_No Orientation on MMS | References to FCHV with no orientation on MMS before | When FCHVs explicitly state they have not received any orientation or training on MMS. | When FCHVs have received any form of orientation or training on MMS. | **User Organization** (Capacity to Implement)_ |
| **8.1 Knowledge and Understanding on MMS** | FCHVs’ knowledge and understanding regarding MMS in the absence of orientation. | When FCHVs discuss what they know or do not know about MMS without prior orientation. | When knowledge is based on formal orientation or training. | **User Organization** (Capacity to Implement)_ |
| **8.2 Experiences with iron tablets** | References to women’s experiences on iron tablets. | When FCHVs or women share stories or feedback about using iron tablets. | When discussing experiences with MMS or other supplements. | **Innovation Attributes**  (Perceived advantages of intervention) |
| **8.3 Reported benefits of Iron tablets** | References to positive outcomes or effects that women have attributed to iron use. | When women or FCHVs report improvements or positive effects from iron tablets. | When benefits are theoretical or not attributed to iron tablets. | **Innovation Attributes**  (Perceived advantages of intervention) |
| **8.4 Side effects and concerns on iron tablets** | References to negative experiences, discomfort, or worries reported by women taking iron | When specific side effects or concerns about iron tablets are mentioned. | When discussing side effects of other supplements or general health issues. | **Innovation Attributes**  (Identifies barriers to adoption) |
| **8.5 Counseling Strategies** | References to specific methods, language, or approaches FCHVs use when explaining iron tab to women. | When FCHVs describe their communication or counseling techniques about iron tablets. | When discussing general education or counseling not specific to iron tablets. | **User Organization Attributes**  (Reflects FCHVs’ operational capacity to deliver the intervention) |
| **8.6 Distribution Channels** | References to where and how iron tab is or should be physically distributed to pregnant women. | When FCHVs describe logistics, locations, or processes for iron tablet distribution. | When discussing distribution of other supplements or non-specific logistics. | **User Organization Attributes**  (User organization logistical capacity to implement) |
| **8.7 Feedback on Iron tablets** | References to women’s f**eedback** on iron tablets | When women provide opinions, suggestions, or reactions to iron tablets. | When feedback is about other supplements or general health services. | **Innovation Attributes**  (End-user feedback on innovation acceptability and adaptability) |
| **8.8 Initial concerns** | References to women’s concerns and initial reactions on iron tablets | When women express worries or hesitations when first introduced to iron tablets. | When discussing concerns after prolonged use or about other products. | **Innovation Attri**butes  (Captures early adoption challenges) |
| **8.9 Service providers** | References to who is responsible for different aspects of iron tab distribution and counseling. | When FCHVs or others mention roles of health workers, supervisors, or other service providers. | When roles are not specified or are about other interventions. | **Resource Team**  (Role of trainers/supervisors in supporting FCHVs) |
| **8.10 Documentation** | References to record-keeping, monitoring, reporting requirements, or data collection related to iron tablets. | When FCHVs discuss forms, registers, or reporting processes for iron tablets. | When documentation is about other interventions or not related to iron tablets. | **Scale-up Strategy**  (Monitoring and evaluation) |
| **8.11 Potential Challenges** | When stakeholders note potential challenges | When FCHVs or others mention anticipated or observed barriers to implementation. | When challenges are not specified or are not related to iron tablet programs. | **User Organization**  (Systemic challenges such as training gaps in the user organization) |
| **8.12 Training needs** | Mentions of training needs, approaches, or programs to equip health workers and FCHVs with knowledge and skills related to iron | When FCHVs or supervisors request or suggest training for iron tablet distribution or counseling. | When discussing training needs unrelated to iron tablets. | **User Organization**  (Implementation capacity) |
| **8.13 Resources required** | References to materials, tools, training, or support systems FCHVs desire to effectively promote iron tab. | When FCHVs request specific resources to help with iron tablet promotion. | When resource needs are not specified or are unrelated to iron tablets. | **Resource Team**  (Materials/support for resource team) |
| **8.14 Good counselling** | References to good counselling and effective messaging on iron tablets importance | When FCHVs or women describe examples of effective counseling or communication about iron tablets. | When counseling is described as ineffective or not related to iron tablets. | **User Organization**  (Institutional capacity to deliver the intervention) |

## 4c. Codebook for Policymaker Key Informant Interviews Using the WHO ExpandNet Framework

### **Overview of the Codebook**

This codebook organizes qualitative data from policymaker interviews into four thematic domains, each mapped to WHO ExpandNet’s scaling principles. It captures critical insights on scaling strategies, implementation challenges, equity considerations, and adaptive governance.

### 1. Codebook Structure

The codebook organizes data into **6 thematic tables**, each containing **parent-child codes** derived from the KII guide and research questions:

| Table | Focus | Key Codes |
| --- | --- | --- |
| 1. Awareness and Advocacy Strategies | Stakeholder engagement, political buy-in, and communication strategies to drive MMS adoption. | Media campaigns (radio/TV/social media) - Policy briefings with evidence summaries - Champion identification (influential policymakers, NGOs) - Addressing cultural myths (e.g., replacing traditional remedies) |
| 2. Transition Implementation | Operational execution of MMS rollout, including capacity building and logistics. | - Budget allocation disputes - Training cascades for health workers - Supply chain integration with ANC services - Decentralized procurement models |
| 3. Monitoring and Adaptation | Systems for tracking progress, addressing challenges, and iterative adjustments. | - Feedback loops (FCHV-led review forums) - Revised HMIS indicators for MMS adherence - Policy harmonization across federal/provincial tiers |
| 4. Equity Considerations | Ensuring equitable access to MMS across socioeconomic and geographic groups. | - Marginalized groups targeting (Dalit/Janajati) - Geographic prioritization (mountain districts first) - Cultural tailoring of IEC materials (local dialects, festivals) - Male gatekeeper engagement strategies |
| 5. Stakeholders | Roles and influence of actors enabling or hindering MMS scale-up. | FCHVs as trusted messengers - Government officials (mayors, district health officers) - Partner NGOs (technical support) - Religious/community leaders (e.g., Maulanas in Terai) - Private sector (supply chain partnerships) |
| 6. Training and Capacity Building | Building technical and operational capacity for sustainable MMS delivery. | - Incentives for health workers - Printed/mass media IEC tools - Financial support mechanisms - Checklists for stock monitoring - Demonstration-based trainings - Orientation sessions for policymakers |

**WHO ExpandNet Framework Alignment**

The codebook maps themes to **5 core components** of the WHO framework to evaluate scalability:

| WHO Component | Explained Description | Example Codes |
| --- | --- | --- |
| 1. Innovation Attributes | Refers to the characteristics and perceived value of the intervention (MMS) itself, including its design, formulation, delivery form, and alignment with local beliefs and practices. This component assesses how acceptable, adaptable, and effective the innovation is in the target context. Key considerations include physical attributes (e.g., pill size, taste), evidence of effectiveness, perceived benefits or risks, and cultural compatibility. | Pill characteristics, perceived effectiveness, cultural acceptability, side effects, packaging, formulation, product superiority, addressing misconceptions |
| 1. User Organization Attributes | Covers the readiness, capacity, and internal processes of the organizations (e.g., health system, government bodies) responsible for adopting and implementing MMS. This includes leadership structures, staff training and motivation, internal communication, resource allocation, and the ability to manage logistics and monitoring. It also addresses how well these organizations can adapt to new protocols and sustain the intervention. | Counseling strategies, training preferences, leadership and governance, supply chain hurdles, implementation challenges, resource allocation, data collection methods |
| 1. Environment Attributes | Encompasses the broader external context-including political, economic, geographic, and socio-cultural factors-that can enable or constrain scale-up. This includes policy environment, community norms, infrastructure, supply chain context, stakeholder engagement, and the influence of external actors (e.g., donors, religious leaders). It also covers barriers such as geographic remoteness, poverty, and local beliefs that affect access and uptake. | Supply chain issues, geographic barriers, political will, community beliefs, economic access, stakeholder engagement, cold-chain gaps, social practices, festival outreach |
| 1. Scale-Up Strategy | Describes the deliberate, systematic approaches and decisions made to expand MMS from pilot or small-scale to broader, sustainable, and institutionalized programs. This includes advocacy and awareness campaigns, phased or targeted rollouts, partnership models, monitoring and evaluation frameworks, and strategies to ensure equity and sustainability. It also covers the mechanisms for adapting and learning during the scale-up process. | Media campaign, phased implementation, partnership models, equity targeting, M&E integration, outreach strategies, multisectoral engagement, cost-effectiveness rationale |
| 1. Resource Team Attributes | Focuses on the individuals and organizations providing technical support, training, capacity building, and ongoing guidance for scale-up. The resource team includes trainers, technical experts, NGOs, private sector partners, and others who help build and sustain the capacity of the user organization. This component covers resource mobilization, development of training materials, provision of incentives, and support for ongoing learning and problem-solving. | Training needs, resource gaps, incentives, technical support, printed materials, mass media for IEC, financial support, efficient manpower, demonstration trainings, orientation sessions |

### 2. Implementation Guidelines in NVivo 14/15

- **Hierarchical Coding:**
  - Create parent codes or WHO ExpandNet components (e.g., *Innovation Attributes*).
  - Nest codebook themes (e.g., *Women’s Experiences*) as child codes.
- **Deductive Coding:** Apply pre-defined codes to transcripts using the codebook’s "When to Use" criteria.
- **Inductive Coding:** Add emergent themes (e.g., "community taboos") as new codes, then map them to frameworks

### Table 1: Awareness & Advocacy Strategies

| **Code** | **Description** | | **Example Quote** | **WHO ExpandNet Component** |
| --- | --- | --- | --- | --- |
| - 1. **StakeholderPercep** | Perceptions about MMS implementation challenges | | "Some communities distrust supplements due to past IFA side effects" | Innovation  Relates to the perceived acceptability of MMS as an innovation |
| **1.2 CommChannelPref** | Preferred communication channels | | "Radio dramas in local dialects reach 80% of our rural population" | Scale-Up Strategy ˃Dissemination Approaches  Relates to the strategic choice for spreading awareness. |
| **1.2a Social Media** | Awareness by the use of social media | | With the rise of social media, almost everyone with a mobile phone has access to it. If we can promote MMS through social media, we can reach and engage a larger portion of the target population. | Scale-Up Strategy ˃Dissemination  Relates to the strategic choice for spreading |
| **1.3 KeyActorMapping** | Critical stakeholders for transition | | "Mayors control health budgets - their buy-in is non-negotiable" | User Organization Attributes ˃Leadership Structure  Relates to institutional leadership roles |
| **1.4 Addressing Misconceptions** | References to misinformation, myths, or misunderstandings about MMS. | | When participants describe incorrect beliefs or fears about MMS. | Innovation Attributes  ˃Cultural compatibility  Relates to the cultural fit of the innovation. |
| **1.4a** **Clear Understanding** | When the stakeholders are clear about the concept. | | There are no such misconceptions. | Innovation  Reflects stakeholders’ grasp of the innovation. |
| **1.5 Knowledge and Understanding (Inductive)** | When stakeholders discuss their general knowledge about MMS or understanding of it | |  | Innovation  Awareness of the innovation’s design. |
| **1.6 Political Will** | References to political will or opposition |  | | Environment > Political context  Relates to political environment |
| **1.7 Policy Formulation and Implementation** |  | At the national level, we focus on policy formulation, strategic planning, technical guidelines, and resource mobilization. | | User Organization > Governance  Institutional capacity for policy adoption |
| **1.8 Social Practices** | When community members have common social behaviors and practices. | In those areas, there are many women who do not visit health institutions for prenatal check-ups. | | Environment > Social Cultural Context  Relates to culture norms in the external environment |

### Table 2: Transition Implementation

| **Code** | **Description** | **Example Quote** | **WHO ExpandNet Component** |
| --- | --- | --- | --- |
| - 1. **SupplyChainHurdles** | Distribution challenges | "Winter road closures disrupt deliveries to mountain districts" | User Organization Attributes ˃ Implementation Capacity  Relates to Institutional capacity to manage supply chains. |
| **2.2 CrossGovtCoord** | Intergovernmental coordination needs | "Federal-provincial health committees must meet monthly" | Scale-Up Strategy ˃Partnership Models  Partnership models for multi-level governance. |
| **2.3 TrainingNeeds** | Capacity building requirements | "Pharmacists need updated dispensing protocols" | Resource Team Attributes  Relates to skill development for implementation support. |
| **2.4 Potential Challenges** | When stakeholders note potential challenges |  | User Organization  Relates to institutional readiness and adaptability |
| **2.4a Potential Challenges_Cost and Resource allocation** | When stakeholders discuss the higher costs of MMS as a potential challenge |  | User Organization  Relates to financial/resource management capacity. |
| **2.4b Potential Challenges_Counselling Skills** | Challenges related to provider or CHW counseling |  | Resource Team  Relates to workforce skill deficiencies. |
| **2.4c Potential Challenges_Efficacy** | When stakeholders question MMS efficacy over IFA | “Some are also questioning whether IFA might be more effective, as the iron content in MMS is slightly lower, which could potentially have a smaller impact” | Innovation Attributes  Relates to the perceived effectiveness of the innovation |
| **2.4d Potential Challenges_Sustainability** | When stakeholders question the potential sustainability of the transition | “People are wondering whether it will be a temporary measure or if it will be implemented long-term.” | Innovation Attributes  Relates to strategic planning for sustainability. |
| **2.4e. Potential Challenges_Side effects** | When stakeholders note specific side effects related to MMS. |  | Innovation Attributes  Relates to the acceptability of the innovation. |
| **2.4f Potential Challenges_Lack of education** |  | Since the Tharu community has relatively less access to education and fewer educated individuals, it might be a bit more challenging to inform them effectively. | Environment  Relates to socio-cultural context affecting uptake. |
| **2.4g Potential Challenges_CrossGovtCoord** | When the respondents note the challenges regrading coordination with multiple donors and government. | If we look at the challenges at the provincial level, one concern is how to coordinate with the central government and communicate with donors. | Scaling up Strategy  Relates to partnership and governance challenges. |
| **2.4h Potential Challenges_Implementation** | When the respondents mention the implementation challenges regarding the program. | Therefore, if this is to be implemented as a national program in the future, it is essential to examine whether such factors might pose challenges regarding acceptability and program implementation. | User organization  Relates to institutional implementation capacity. |
| **2.4i Potential Challenges_Program Acceptability** | When the community have concerns with the acceptance. | When a program is introduced at the community level, we must assess what | Innovation  Relates to the socio-cultural acceptability of the innovation. |
| **2.4j. Potential Challenges_Cultural Barriers** | When the cultural practices negatively impact the implementation process | Language barriers must also be addressed by ensuring that messages come from within the community in their own language and cultural context. | Environment  Relates to the culture norms in the external context. |
| **2.4k. Potential Challenges_Lack of awareness** | When lack of awareness impacts the transitional implementation | if people are unaware of why they need to consume them that can also be a reason for non-consumption. | Innovation Attributes  Awareness of innovation benefits. |
| **2.5 Reasons for Transition** | When respondents discuss the various reasons for the transition |  | Scaling-up Strategy  Strategic rationale for scaling. |
| **2.5a. Reason_HealthOutcomes** | When respondents say that emphasizing health outcomes is a key message. |  | Innovation Attributes  Perceived benefits of the innovation. |
| **2.5b. Reason_CostEffectiveness** |  |  | Scaling up Strategy  Resource optimization strategy |
| **2.5c Reason_Product Superiority** | When respondents say that emphasizing the superiority of MMS over IFA is a key message. |  | Innovation attributes  Relative advantage of the innovation |
| **2.5d Reason_Reduced Side Effects** | Fewer side effects |  | Innovation attributes  Relative advantage of the innovation |
| **2.6 Rollout Recommendation** | When stakeholders provide recommendations on how to best rollout the transition | “Pregnant women must visit the health post to take IFA, so it would be much better if counseling is provided when they first receive it.” | Scaling-up strategy  Strategic implementation approaches. |
| **2.6a Recommendation_Good counselling provider** | When stakeholders mention health provider counseling as important for successful rollout | “Patients tend to trust whatever health workers say. It all depends on how well the health workers are able to explain things.” | Resource Team  Workforce skill development |
| **2.6b Recommendation_Community and family Engagement (Inductive)** | When community engagement is indicated as important for the transition | “If they can be properly informed, the implementation of this initiative will be more successful.” | Scale-up strategy  Socio-cultural engagement. |
| **2.6c Recommendation_Awareness of Benefits** | When awareness of benefits and correct information is noted as important for the transition | “Without the right information, people won’t be motivated to learn more. But when they are given prior information about the benefits for both the baby and the mother, they will find it easier to understand and accept.” | Scale-up strategy  Information and dissemination messaging to improve awareness and knowledge. |
| **2.6d Recommendation_Coordination** | When coordination is noted as important for the transition |  | Scaling-up Strategy  Partnership models |
| **2.6e Recommendation_ Supply chain forecasting and commodity management** | When issues related to supply chain, logistics, and forecasting are mentioned as important for the transition |  | User organization  Institutional operational capacity |
| **2.6f Recommendation_Outreach** | When outreach is noted as a key strategy for successful transition |  | Scaling-up strategy  Dissemination approaches |
| **2.7 Awareness Strategies** | When providing specific awareness raising strategies in the community (i.e. mobilizing FCHVs, TV, mass media, radio spots), etc. |  | Scale-up strategy  Dissemination approaches |
| **2.7a. Awareness_ Event** | When holding an event is noted as an awareness strategy |  | Scale-up strategy  Targeted advocacy |
| **2.7b. Awareness_Social Media** | When social media is noted as an awareness strategy |  | Scale-Up Strategy ˃Dissemination  Relates to the strategic choice for spreading  [Note: same as 1.2] |
| **2.7c. Awareness_Festivals** | When outreach using festivals are mentioned as an awareness raising platform or strategy. | “Resources and means can be allocated for some festivals. For example, among the Tharus, if someone in their community is getting married, the entire community goes and participates.” | Scaling-up Strategy ˃Dissemination |

### Table 3: Monitoring & Adaptation

| **Code** | **Description** | **Example Quote** | **WHO ExpandNet Component** |
| --- | --- | --- | --- |
| **3.1. M&E_Integration** | Monitoring system proposals | "Modify HMIS to track MMS stockouts in real-time" | Scale-Up Strategy ˃Evaluation Framework  Strategic monitoring systems are part of scaling-up evaluation. |
| **3.2. Adaptive Mechanisms** | Responsive adjustment strategies | "Quarterly review forums with FCHVs ensure course correction" | Innovation Attributes ˃ Adaptability  Reflects the innovation’s capacity to adapt to feedback. |
| **3.3. Review meetings_Feedback Loops** | When review meetings are noted as a strategy to improve overall quality of service delivery |  | User organization  System readiness for feedback in M&E system. Internal system structures for ongoing improvement |
| **3.4 Data Collection Methods** | Mentions of monitoring tools or approaches |  | User organization  Organizational ability to measure and monitor impact |

### Table 4: Equity Considerations

| **Code** | **Description** | **Example Quote** | **WHO ExpandNet Component** |
| --- | --- | --- | --- |
| **4.1 GeoAccess** | Remote area challenges | “If we look at the villages, there are also very remote places in the municipalities. For that, we may have to go door to door. Sometimes there are situations where there is no access to some resources.” | Environment Attributes ˃ Geographic Context  External geographic and infrastructural barriers impacting equity in access |
| **4.1a Marginalized Groups targeting** | Targeting marginalized groups | “From an economic perspective, there are rural and remote areas in the region, but if the benefits of this initiative are clearly explained, I don't believe it will have a significant financial impact.” | Scaling-up strategy  Strategic focus on ensuring scale-up reaches marginalized populations. |
| **4.2 EconAccess_SES** | Poverty-related barriers or General issues related to education and SES | "Daily wage earners can't attend daytime trainings" | Environment Attributes  Socio-economic environment affecting equitable participation and access. |

### Table 5: Community Stakeholders

| **Code** | **Description** | **Example Quote** | **WHO ExpandNet Component** |
| --- | --- | --- | --- |
| **5.1. Stakeholders_FCHV** | When FCHVs are noted as a key stakeholder for the transition | “Female health volunteers play a significant role in this as well. What they say is often taken seriously by the community.” | Resource team  Relates to FCHVs are a core part of the resource team providing technical support and facilitating scale-up at the community level. |
| **5.2. Stakeholders_Govt officials** | When government officials (e.g. mayors or chairperson, district officials, public health officials) are noted as a key stakeholder for the transition |  | User Organization  Relates to Government officials represent the leadership and institutional capacity responsible for adoption and implementation. |
| **5.3. Stakeholders_Private Sector** | When private sector mentioned as a key stakeholder for the transition |  | Resource team  The private sector can provide technical, logistical, and financial resources for scale-up. |
| **5.4 Stakeholders_Partner NGO** | When partner NGO mentioned as a key stakeholder for the transition |  | Resource team  NGOs are part of the resource team, offering technical assistance, capacity building, and advocacy. |
| **5.5. Stakeholders_Health providers** | When health providers are mentioned as a key stakeholder for the transition |  | Resource Team  Health providers are frontline members of the resource team, delivering services and information. |
| **5.6. Stakeholders_Multisectoral Engagement** | When multiple sectors are noted as necessary for the transition |  | Scaling-Up Strategy  Multisectoral engagement is a strategic approach to ensure sustainability and broad-based support for scale-up. |
| **5.7 Stakeholders_Community Religious leaders** | When trusted community leaders  Or religious leaders are noted as necessary for the transition | If an influential religious figure, such as a Maulana, tells the community that a particular food supplement is beneficial, they are more likely to accept it. | Scale-up Strategy  When mention leveraging community and religious leaders shape the socio-cultural environment and influence community acceptance of the innovation. |

### Table 6. Training and Capacity Building

| **Code** | **Description** | **Example Quote** | **WHO ExpandNet Component** |
| --- | --- | --- | --- |
| **6.1 Resources Needed** |  |  | **Resource Team**  Resource Team is responsible for mobilizing and providing the necessary resources for scaling. |
| **6.1a. Resources_Incentives** | When incentives are needed to motivate performance and ensure a successful transition |  | **Scaling up Strategy**  When Incentives are noted as part of scale up strategy and need resource mobilization and staff motivation managed by the resource team. |
| **6.1b. Resources_Printed materials** | When printed media is noted as important for IEC and awareness-raising |  | **Scaling-up Strategy**  When IEC materials are tools are noted ad needing to be provided by the resource team for training and awareness. |
| **6.1c. Resources_mass media** | When mass media is noted as an important IEC strategy and for training |  | **Scaling-up Strategy**  Mass media for IEC and training is coordinated by the resource team to build capacity and awareness. |
| **6.1d Resources_Checklists** | When facility checklists (e.g. inventory form, service checklists) are noted as needed for the  transition |  | **Resource team**  When checklists or implementation tools developed and distributed by the resource team. |
| **6.1e Resources_Financial support** | When the financial support and funding is required for the transition of MMS rather than incentive |  | **Resource Team**  When financial support is a core responsibility of the resource team for sustainable scale-up. |
| **6.1f Resources_Efficient Manpower** | When the technical personnel and more trained staff are recommended for the transition | To increase monitoring, we need to address this gap by involving more trained staff | **Resource Team**  Ensuring adequate and skilled manpower is a direct function of the resource team. |
| **6.2 Training Needed** |  |  | **Resource Team**  Training and capacity building are primary functions of the resource team. |
| **6.2a. Training_Demonstrations** | When training using demonstrations are recommended |  | **Resource Team**  Demonstrative training methods are part of resource team’s approach to effective capacity building. |
| **6.2b. Training_Orientation** | When orientation is recommended | An orientation session would be sufficient because they need to have basic knowledge about MMS | **Resource Team**  Orientation is a key training activity facilitated by the resource team for knowledge transfer. |
